# ﻿An updated checklist of vascular plants of Myanmar

**DOI:** 10.3897/phytokeys.261.154986

**Published:** 2025-08-11

**Authors:** Ye Lwin Aung, Mung Htoi Aung, Yunhong Tan, Xiaohua Jin

**Affiliations:** 1 State Key Laboratory of Plant Diversity and Specialty Crops, Institute of Botany, Chinese Academy of Sciences, Beijing 100093, China Chinese Academy of Sciences Beijing China; 2 University of Chinese Academy of Sciences, Beijing 100049, China University of Chinese Academy of Sciences Beijing China; 3 Forest Department, Forest Research Institute, Yezin, Nay Pyi Taw 1505204, Myanmar Forest Research Institute Nay Pyi Taw Myanmar; 4 AgroParisTech, 14 Rue Girardet, 54000 Nancy, France AgroParisTech Nancy France; 5 Southeast Asia Biodiversity Research Institute, Chinese Academy of Sciences, Yezin, Nay Pyi Taw 1505204, Myanmar Chinese Academy of Sciences Nay Pyi Taw China; 6 Center for Integrative Conservation & Yunnan Key Laboratory for the Conservation of Tropical Rainforests and Asian Elephants, Xishuangbanna Tropical Botanical Garden, Chinese Academy of Sciences, Mengla, Yunnan 666303, China Chinese Academy of Sciences Yunnan China; 7 Yunnan International Joint Laboratory of Southeast Asia Biodiversity Conservation, Mengla, Yunnan, 666303, China Yunnan International Joint Laboratory of Southeast Asia Biodiversity Conservation Yunnan China; 8 China National Botanical Garden, Beijing 100093, China China National Botanical Garden Beijing China

**Keywords:** Biodiversity conservation, botanical collections, herbarium specimens, Myanmar flora, Southeast Asia

## Abstract

Myanmar is one of the Southeast Asian countries where biodiversity richness is very high as well as under various anthropogenic threats. Its broad latitudinal range, heterogeneous topography, and tropical monsoonal climate make the country exceptionally rich in plant biodiversity. However, botanical exploration in Myanmar still lags, hindering a full understanding of the floristic diversity of the country and leading to a large gap in taxonomic knowledge of its flora. The latest checklist of Myanmar plants (including 11,800 species) was published over 20 years ago and clearly needs significant and comprehensive revisions to be in line with modern taxonomic classification systems. In this regard, the present study investigated the species richness of Myanmar flora based on herbarium specimens, taxonomic literature, and online databases. Therefore, it resulted in an updated checklist consisting of 14,020 species in 2,701 genera and 292 families of vascular plants known from Myanmar. Among them, there are 13,314 native species and 706 introduced species in Myanmar. In Myanmar, there are 864 endemic species which need proper conservation actions. In comparison, the number of species in the updated checklist has increased by 2,220 species more than those of the previous checklist, mainly due to the discovery of new species and new records for the country. The updated checklist has been taxonomically verified with voucher specimen-based comparisons, which will be useful for subsequent analyses of biodiversity research and conservation action.

## ﻿Introduction

**Figure 1.**
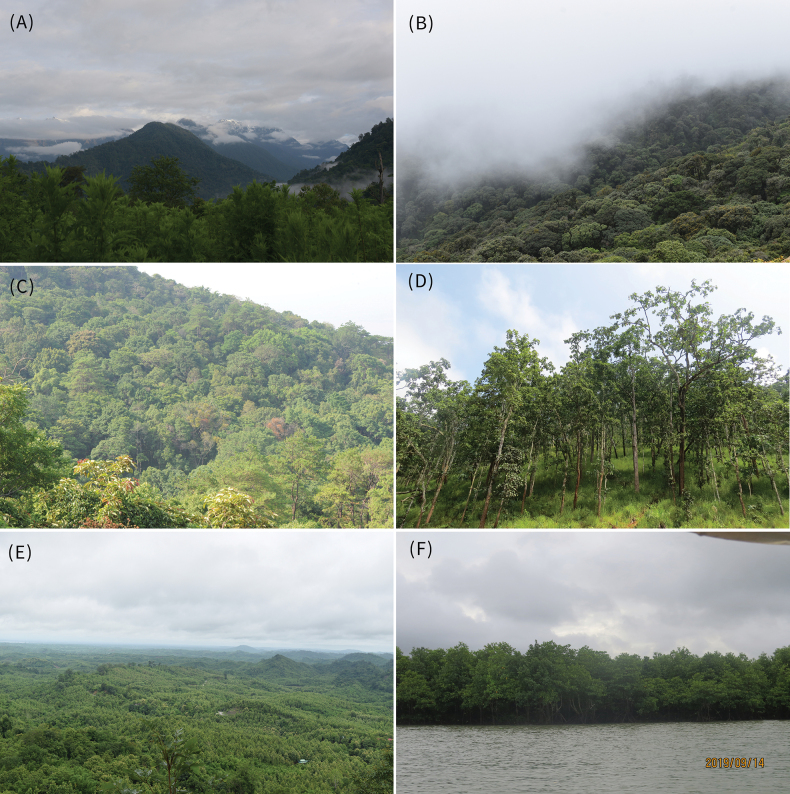
Different Forest Ecosystems of Myanmar which are home to various species of fauna and flora. A. Subtropical montane forest in Kachin State; B. Tropical montane forest in Chin State; C. Tropical broadleaved forest in Mandalay Region; D. Dipterocarp forest in Kayah State; E. Tropical moist and dry deciduous forest in Bago Region, and F. Coastal Mangrove forest in Ayeyarwaddy Region. Photo (A) by Xiaohua Jin, and Photo (B–F) by Ye Lwin Aung.

**Figure 2.**
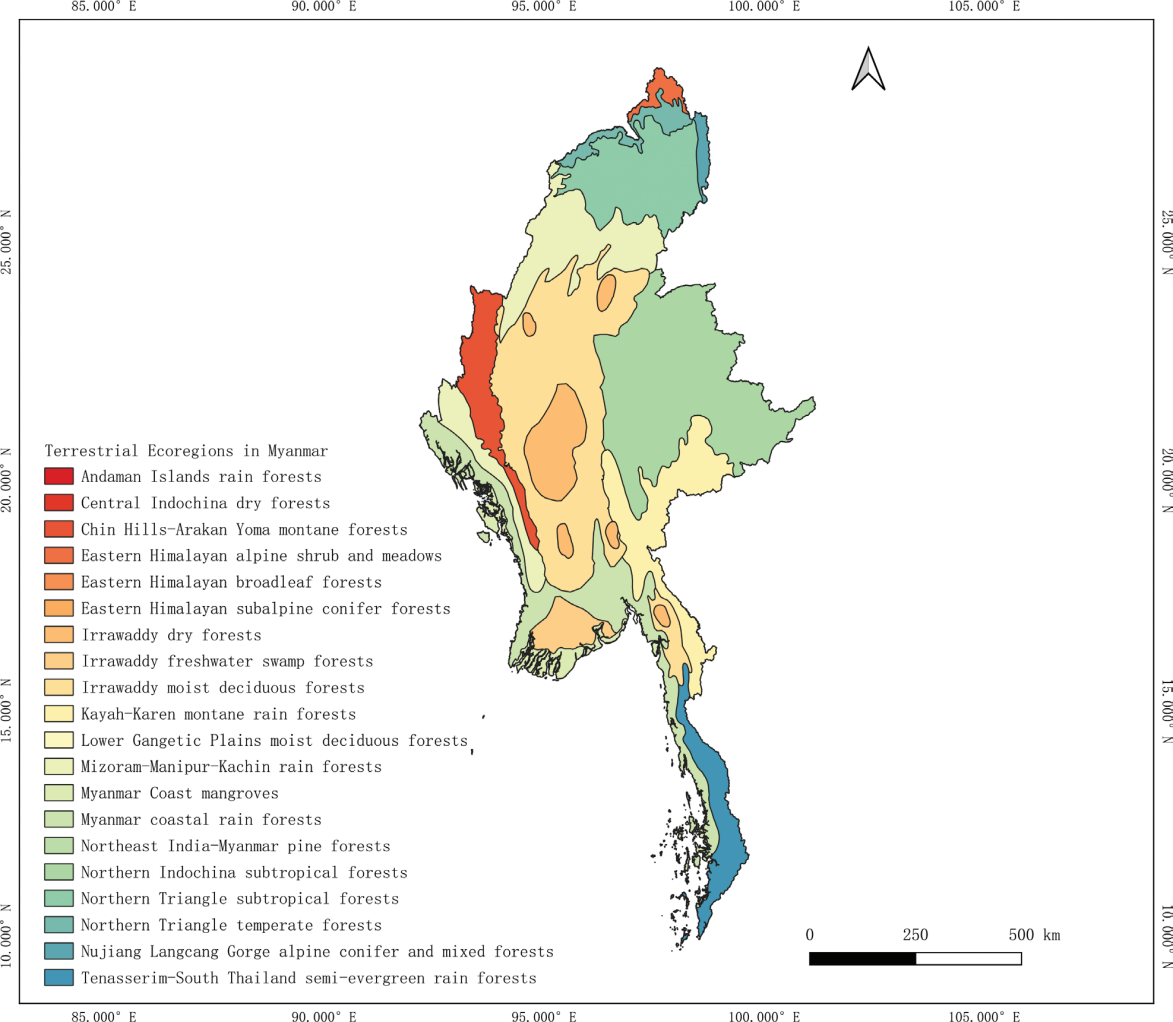
Different terrestrial ecoregions in Myanmar, mainly based on the global terrestrial ecoregion delineation (Olson et al. 2001).

**Figure 3.**
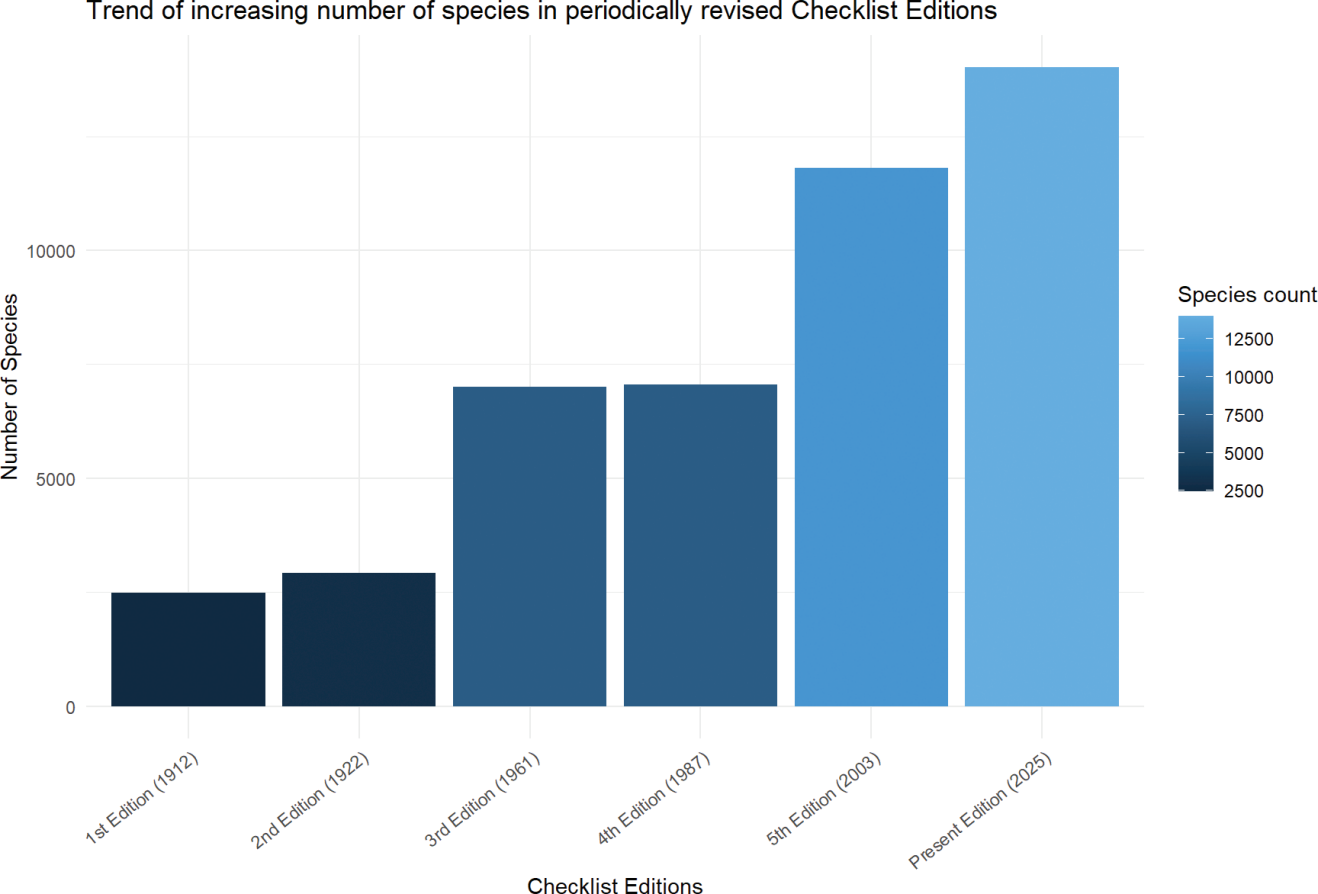
Trend of increasing number of species in the periodically revised checklist editions.

Myanmar is situated in Southeast Asia and included in the Indo-Burma global biodiversity hotspot, with high species richness and diversity (Myers et al. 2000; Mittermeier et al. 2011). Because of its broad latitudinal range (tropical to subtropical) as well as topographical (mostly mountainous) and climatic (monsoonal) factors, numerous types of ecosystems are found in Myanmar, from southern tropical evergreen rainforest ecosystem to northern subtropical alpine meadow through central dry deciduous forests (Fig. 1). As for area-based conservation approach of global ecoregion delineation, there are 20 terrestrial ecoregions (Fig. 2) and 3 marine ecoregions in Myanmar which serve as baseline biodiversity data for spatial conservation prioritization schemes for the country (Olson et al. 2001; UNDP and SCBD 2021). In addition, a recent study by Murray et al. (2020) identified 64 different ecosystem types in Myanmar, which are in need of urgent conservation actions (Murray et al. 2020). Biodiversity conservation actions should be accelerated to secure the sustainable development of the country in the long term.

The plant biodiversity of Myanmar is very rich but very poorly known due to insufficient botancial survey and research in the past decades, resulting in a large gap of knowledge about the flora of the country (Kress et al. 2003). It is evident that recent botanical explorations have resulted in the discovery of many new species to science as well as new records for Myanmar flora, suggesting that the underexplored regions of the country host many yet undescribed species as well as not-yet-recorded species, families or even orders of plant biodiversity. In this regard, a recent study also highlighted that Myanmar is one of the countries within the plant diversity blindspots, where it is predicted to contain possibly the most undescribed species and not-yet-recorded species in the respective countries (Ondo et al. 2024).

Historically, floristic studies in Myanmar dated back to the time of the British colonial regime. Significant floristic documents on the regional flora, included Kurz’s “The Forest Flora of British Burma” (1877) and Hooker’s “Flora of British India” (1894). It is noted that the past botanical explorations and documentations had mainly focused on tree species for commercial timber production.

The first checklist of plants of Myanmar was compiled by J.H. Lace in 1912, including 2,483 species known from Myanmar. In the course of taxonomic enumeration of plant species of Myanmar, the number of recorded species has increased based on available literature and herbarium collections (Kurz 1877; Hooker 1894; Lace 1912; Rodger 1922; Hundley and Ko 1961; Hundley 1987; Kress et al. 2003). At the start of the 21^st^ century, the latest edition of a checklist of plants of Myanmar was published, including approximately 11,800 species in 2,371 genera and 273 families of angiosperms and gymnosperms (Kress et al. 2003).

The latest checklist is now over 20 years old and has served well as the baseline data for the plant biodiversity research and conservation in Myanmar (Kress et al. 2003). Since that publication advances in plant taxonomy and systematics have provided new insights into the classification systems of plant biodiversity, resulting in an updated classification of Angiosperms (Byng et al. 2016), a new classification of Gymnosperms (Christenhusz et al. 2011; Yang et al. 2022), and additional taxonomic information on ferns and lycophytes (PPGI 2016). In addition, the advances in plant systematics have resulted in updated classification systems for specific plant groups, such as orchids, grasses, legumes, and others (Chase et al. 2015; Larridon et al. 2021; Bruneau et al. 2024).

In the review on the checklist of Kress et al. (2003) and taxonomic works by the subsequent researchers (Murata et al. 2010; de Kok 2012; Kazumi et al. 2017; Tan et al. 2017; Jin and Mint 2018; Jin et al. 2018; Kang et al. 2018; Ruchisansakun et al. 2018; Hughes et al. 2019; Tanaka and Aung 2019; Aung et al. 2020; Dong 2020; Ormerod et al. 2021; Tanaka et al. 2022b; Kurzweil et al. 2023), species names have required significant revisions with the modern taxonomic treatments due to: (1) recent discovery of new species and new records from Myanmar, which possibly lead to new additions to flora of Myanmar, (2) taxonomic changes largely occurred in the checklist of Kress et al. (2003) which possibly lead to new combinations or alignments among various taxa including families, genera and species of the checklist, and (3) the linkage between taxonomic status and conservation assessment, which plays a vital role in the conservation assessment for a given plant species at national as well as regional scale (Antonelli et al. 2023).

Recently, new floristic elements of Myanmar, including new records and new species, have been discovered in various ecosystems of Myanmar. In this regard, recent botanical survey programs resulted in discoveries of 193 species new to science as well as 347 species newly recorded from Myanmar within the period from 2000 to 2019 (Yang et al. 2020), leading to many new additions to the flora (Fujiwara et al. 2022; Hein et al. 2022; Tanaka et al. 2022a; Floden et al. 2023; Maw et al. 2023; Jung et al. 2024; Naive et al. 2024). In addition, recent taxonomic work on some plant groups of Myanmar has resulted in publication of respective species checklists, for example, family Orchidaceae, genus *Agapetes* (Ericaceae), genus *Begonia* (Begoniaceae), genus *Polygonatum* (Asparagaceae), genus *Strobilanthes* (Acanthaceae), on genus *Habenaria* (Orchidaceae) and family Balsaminaceae, and so on (Ruchisansakun et al. 2018; Hughes et al. 2019; Aung et al. 2020; Ormerod et al. 2021; Tong et al. 2022; Wood et al. 2022; Floden et al. 2023; Kurzweil et al. 2023).

Updated classification systems of angiosperms, gymnosperms and pteridophytes (Christenhusz et al. 2011; Byng et al. 2016; PPGI 2016; Yang et al. 2022) require a review on family circumscriptions, taxonomic placement of genera, and generic or species name changes in the checklist of Kress et al. (2003). For example, some families are subsumed into a single large family; some genera of a given family have been transferred into another family; and some generic or species names changed into various ways, such as combinations (e.g., heterotypic synonyms or homotypic synonyms) or separations (e.g., raised into upper ranks [genus or species] or reduced into lower ranks [subspecies or varieties]) according to the modern classification systems.

Examples include (1) family Caesalpiniaceae, family Fabaceae and family Mimosaceae are combined into a single large family Fabaceae; (2) family Aceraceae is subsumed into family Sapindaceae; (3) family Alangiaceae is subsumed into family Cornaceae; and (4) family Flacourtiaceae is subsumed into family Salicaceae (Byng et al. 2016). In addition, the genera *Premna*, *Tectona* and *Vitex* in family Verbenaceae have been transferred into family Lamiaceae (POWO 2024), and the orchid genera *Drymoda*, *Ione*, *Monomeria*, *Sunipia* and *Trias* have been merged into the genus *Bulbophyllum*, leading to nomenclatural changes in *Bulbophyllum* (Pridgeon et al. 2014; POWO 2024). The current checklist of plants of Myanmar (Kress et al. 2003) is clearly in need of revisions to be compatible with modern knowledge of the classification systems of plant biodiversity.

The checklist of Kress et al. (2003) also did not include pteridophytes, reflecting a significant research gap on these taxonomic groups in Myanmar (Kress et al. 2003; Khine et al. 2017). Taxonomic studies on pteridophytes of Myanmar dated back to 1940s, with the publication of comprehensive list of Myanmar ferns by Dickason (1946) and a few studies in the past decades. At the start of the 21^st^ century, the taxonomic studies on Myanmar ferns became revitalized with the publication of new species and new records, conservation assessments and fieldwork-based species dataset, leading to contributions to taxonomic knowledge on ferns of Myanmar (Dickason 1946; Khine et al. 2017; Nwe et al. 2019; Khine and Schneider 2020a, 2020b; Fujiwara et al. 2022).

Moreover, bryophyte flora of Myanmar, an understudied taxonomic group, was also not included in the checklist of Kress et al. (2003). Although the floristic studies on bryophyte group of Myanmar dated back to the 1940s, there were very few studies on this taxonomic group in the past decades (Bartram 1943; 1954). Recent years have seen the discovery of new species and new records for Myanmar bryophyte flora, but it is still very far from a complete understanding of this taxonomic group in Myanmar (Potemkin and Müller 2020; Muller and Koponen 2022; Inoue and Aung 2023). Many more taxonomic studies on this taxonomic group are needed in Myanmar.

The present study did not include this taxonomic group because there were some limitations on the availability of taxonomic data on Myanmar bryophyte flora. It is expected that Myanmar bryophyte flora might receive the taxonomic treatments from local taxonomists as well as international taxonomists to better understand and document the bryophyte species richness of the country.

As for floristic studies on Myanmar flora, many herbarium collections were deposited at global and national herbaria, but these collections were not sufficient to cover the whole floristic diversity of the country in terms of taxonomic, temporal and spatial coverage. In addition, further examinations of these collections are still needed to reach the species level identification and perhaps these collections hold many undescribed species, evidenced by recent descriptions of new species from these old collections (Roberts et al. 2008; Ormerod and Wood 2010; Tanaka and Peng 2016; Ormerod and Kurzweil 2020; Aubriot and Knapp 2022; Yukawa et al. 2022).

Although collection efforts are obviously related to species discovery rate, the collection efforts are largely uneven in Myanmar, hindering the understanding on the species diversity and distribution patterns of Myanmar flora. In this regard, the species distribution modelling can be considered as one of the solutions to such challenges of insufficient botanical collections in Myanmar. The well-developed species distribution models can be used for spatial conservation planning schemes as well as prioritization of botanical collection areas. In fact, it is still challenging how to fine-tune the relevant parameters to reach sound and robust species distribution models. However, the species distribution models can be well-developed by using statistical methods as well as machine learning methods based on available species data and environmental datasets (Cai et al. 2023).

Taxonomic data plays a key role in biodiversity conservation planning and implementation at the national as well as regional and global scale (Sandall et al. 2023). In this regard, the main objectives of the present study were set as follows; (1) to provide the updated checklist of Myanmar vascular plant species for floristic studies and biodiversity conservation in Myanmar, (2) to highlight the species richness of Myanmar flora and its conservation actions, and (3) to propose the future directions for plant biodiversity research and conservation in Myanmar.

Therefore, the present study was conducted to investigate the species richness of Myanmar flora mainly based on all available data including herbarium specimens, taxonomic literature and online databases. The updated taxonomic names will be consistent with the species occurrence data or conservation status information in various global databases such as GBIF, IUCN Red List, CITES, WFO, POWO and so on (Sandall et al. 2023). The updated checklist of Myanmar flora provided here will serve as the baseline data going forward for biodiversity research and conservation in Myanmar.

## ﻿Material and methods

### ﻿Study area

Myanmar is located between 9°32'N and 28°31'N, and 92°10'E and 101°11'E, having total area of 676,577 km^2^. Myanmar is one of the Southeast Asian countries and it is bordered by China to the north and northeast, Laos and Thailand to the east, India, Bangladesh and Bay of Bengal on the west, and the Andaman Sea to the south. Myanmar is a tropical monsoon country and its topography is generally mountainous in its eastern, northern, western regions, with central plain region and southern coastal region (Kress et al. 2003; FD 2020). There are 15 administrative provinces in Myanmar, including Nay Pyi Taw Union Territory, seven Regions (Ayeyarwaddy, Bago, Magway, Mandalay, Sagaing, Tanintharyi and Yangon) and seven States (Chin, Kachin, Kayah, Kayin, Mon, Rakhine and Shan). The vegetation of the country consists of tropical lowland evergreen rain forest in the south, tropical hilly evergreen forest in the east, north and west, and central dry and moist mixed deciduous forests. The forest cover area of Myanmar is 42.19% of its total land area according to the Global Forest Resource Assessment Report (FAO 2020). Reserved Forest (RF), Protected Public Forest (PPF) and Protected Areas are legally designated across the country, currently amounting up to 17.76%, 8.15% and 6.43% of the country’s total area, respectively. Myanmar Forest Department is mainly responsible for the management of these legally designated forest land as well as inland wetland, coastal and marine protected areas of the country.

### ﻿Data sources

There are three main data sources for compilation of the updated checklist dataset; (a) herbarium collections which consist of the herbarium collections kept at herbaria (PE and RAF) and the digital herbarium collections accessed on the online herbaria, (b) the taxonomic literature which mainly consists of the literature on new species description, new record discovery and species checklists of Myanmar flora, and (c) the online databases which consist of GBIF and WCVP database (dataset extracted via rWCVP R package).

### ﻿Herbarium collections

#### ﻿Botanical survey

In order to investigate the plant biodiversity of Myanmar, the botanical explorations were conducted in different ecosystems of Myanmar. During 2014–2024, the fieldwork activities were conducted in various protected areas established across Myanmar, namely Hponkanrazi Wildlife Sanctuary and Hkakaborazi National Park of Kachin State, Mt. Zwekabin of Kayin State, Popa Mountain Park of Mandalay Region, Nat Ma Taung National Park and Bwe Par Taung National Park of Chin State, Alaungdaw Kathapa National Park of Sagaing Region, Taunggyi Bird Sanctuary and Loimwe Protected Area of Shan State, and Tanintharyi Nature Reserve of Tanintharyi Region, resulting in the collections of ca. 3,000 herbarium specimens which were kept in herbarium (PE) and herbarium (RAF) (Herbarium codes follow Thiers, updated continuously).

#### ﻿Online herbaria

In order to broaden understanding of the floristic diversity of Myanmar, the digital herbarium specimens were accessed from the online herbaria. All available datasets of herbarium specimens and specimen photographs were examined to enumerate the number of species and to investigate the species occurrences in Myanmar. The following are specimen records examined at online herbaria: BM (2,738 records), A (450 records), AMES (224 records), ECON (5 records), FH (54 records), GH (126 records), K (18,327 records), NY (2,759 records), P (506 records), E (23,302 records), US (6,412 records), B (37 records), GZU (102 records), HAL (8 records), HBG (66 records), JE (5 records), PRC (8 records), W (93 records), WU (9 records), and L (4,673 records) (Harvard University Herbaria and Libraries 2024; Natural History Museum 2024; New York Botanical Garden 2024; Royal Botanic Garden Edinburgh 2024; Smithsonian National Museum of Natural History 2024; The BioPortal of Naturalis Biodiversity Center 2024; The Vascular Plants Muséum National d’Histoire Naturelle 2024; JACQ 2025; Royal Botanic Gardens Kew 2025).

Moreover, the digital herbarium specimens were also accessed from country-specific databases, namely (a) Flora of Myanmar Database (FOMDB 2025) which contains collections kept at herbaria (TNS, TI and RAF) and (b) Myanmar Vascular Plants Database (MBKDatabase 2025) which contains collections kept at herbarium (MBK). Particularly, only the specimen records with species level identification and locality information were selected and transformed into the specific formats to incorporate into the updated checklist. It is noted that some duplicate herbarium specimens can be found across various herbaria, possibly due to their specimen exchange programs.

### ﻿Taxonomic literature review

Besides the examination of herbarium specimens and field observations, the plant species information was extracted from taxonomic literature on new species description, new records discovery, taxonomic revisions, and species checklists of Myanmar flora. The search keywords (“flora” or “new species” or “new records” or “checklists” and “Myanmar”, or “Burma”, or “Southeast Asia”) were used to find the relevant taxonomic literature on flora of Myanmar in the major academic databases such as Web of Science, Google Scholar, ResearchGate and so on.

### ﻿Online databases

In addition to the online herbaria, the species datasets were downloaded from online databases such as GBIF (Global Biodiversity Information Facility). As for GBIF, there are two different datasets for plants of Myanmar, particularly (1) the dataset of higher plants in Myanmar (Aung and Xu 2023) and, (2) the species occurrence dataset of plants in Myanmar (GBIF.org 2025). The species dataset for Myanmar was extracted from the World Checklist of Vascular Plants (WCVP) by using rWCVP R package (Brown et al. 2023). All the downloaded and extracted datasets were verified and transformed into the specific formats to incorporate into the updated checklist of vascular plant species of Myanmar.

### ﻿Data compilation

Basically, there are two primary criteria for taxon inclusion or exclusion for the updated checklist compilation, namely (1) accepted taxonomic status: a given taxon must be taxonomically accepted, and (2) reliable occurrence status: a given taxon must have sound evidence of occurrence in Myanmar. As for family level taxonomic circumscriptions, we followed the APG IV for Angiosperms (Byng et al. 2016), updated classification system for Gymnosperms (Christenhusz et al. 2011; Yang et al. 2022) and PPG I for pteridophytes (PPGI 2016).

As for generic delimitations and species delimitations, we usually consulted the updated classification systems for specific plant groups such as family Orchidaceae (Chase et al. 2015, 2020, 2021; Ng et al. 2018), family Cyperaceae (Larridon et al. 2021) and so on. Particularly, the World Flora Online taxonomic backbone was followed for the taxonomic names and their respective taxonomic status (WFO 2024). All species datasets derived from three main sources were compiled into the updated checklist of vascular plants of Myanmar, following the modern taxonomic classification systems for angiosperms, gymnosperms and pteridophytes.

The data quality and availability usually vary from species to species, for example, some species have many herbarium specimen records while some species have very few herbarium specimen records or even lacking in some cases. Therefore, a given species must be not only taxonomically accepted but also have evidence of occurrence records in Myanmar, sourced from either one or all three main sources.

If there were some issues for inclusion or exclusion decisions, such as taxonomic conflicts between two or more classifications and the vague occurrence of untraceable species, the taxonomic status of a given species was usually decided mainly based on the two reliable databases such as POWO and WFO because these databases are regularly fed with updated systematics and taxonomic literature. Although there are a few discrepancies between them, such discrepancies might not largely affect the inclusion or exclusion decisions for most species. The occurrence status of a given species was usually determined by reviewing the species occurrence information.

International Plant Names Index (IPNI) database was usually consulted for taxonomic name standardization, particularly in case of a resolution to the unclear taxonomic names (IPNI 2024). In addition, all the taxonomic names of the updated checklist dataset were standardized by using WorldFlora R package in R software as well as using an online tool, namely Taxonomic Name Resolution Service V5.3.1 (TNRS), setting both tools at WFO taxonomic backbone to be consistent in terms of taxonomic names (Kindt 2020; R Core Team 2024; TNRS 2025). The taxonomic name standardization makes the updated checklist dataset ready for the potential research uses in biodiversity conservation as well as ecological studies on plant biodiversity of Myanmar.

### ﻿Verification of the checklist dataset

The updated checklist dataset was verified with the reliable online databases such as World Flora Online (WFO), Plants of the World Online (POWO) and International Plant Names Index (IPNI) (IPNI 2024; POWO 2024; WFO 2024). By using rWCVP R package and LCVP R package, the taxonomic name matching of the updated checklist dataset was conducted against WCVP database and LCVP database, resulting in 98% of the taxa names well matched to WCVP database and 96% of the taxa names well matched to LCVP database (Freiberg et al. 2020; Brown et al. 2023).

In addition, the species list for Myanmar was extracted from BIEN database (the Botanical Information and Ecology Network) by using BIEN R package and then the updated checklist dataset was cross checked with the BIEN dataset (including 8,236 taxa), resulting in the fact that 88% of the taxa names were shared between the two different datasets (Enquist et al. 2016; Maitner et al. 2018).

Moreover, the globally assessed vascular plant dataset for Myanmar was downloaded from IUCN Red List database, amounting to 2,436 species known to occur in Myanmar (IUCN 2025). The Red List dataset was cross checked with the updated checklist dataset, resulting in the fact that 96% of the taxa names of the Red List dataset have been included in the updated checklist dataset.

Therefore, the updated checklist dataset was taxonomically verified according to the modern classification systems and the taxa of the updated checklist dataset shared largely with those of the various global online databases such as WCVP, LCVP, BIEN and so on.

All the tasks of data preparation, processing, compilation, and graphical visualizations were conducted in R version 4.4.2 with relevant packages such as "*dplyr, data.table, writexl, tidyverse, ggplot2*" and their associated packages (Wickham 2016; Wickham et al. 2019; Wickham et al. 2023; Ooms 2024; R Core Team 2024; Barrett et al. 2025). All maps were created in QGIS version 3.40.4 (QGIS.org 2025).

## ﻿Results

### ﻿Species richness of Myanmar flora

The present study results in an updated checklist consisting of 14,020 species in 2,701 genera and 292 families of vascular plants known from Myanmar (Table 1). Among them, there are 13,314 native species and 706 introduced species in Myanmar. As for occurrence type, the genera composition can be categorized into three main groups, namely (a) genera with both native and introduced species (9%), (b) genera with only native species (83%), and (c) genera with only introduced species (8%). In addition, family composition can also be categorized into three main groups, namely (a) families with both native and introduced species (34%), (b) families with only native species (64%), and (c) families with only introduced species (2%).

**Table 1.** The summary statistics of vascular plant species in Myanmar.

No.	Major Group	Number of Families	Number of Genera	Number of Species
1.	Angiosperm	248	2,562	12,984
2.	Gymnosperm	9	27	82
3.	Pteridophyte	35	112	954
	Total	292	2,701	14,020

**Figure 4.**
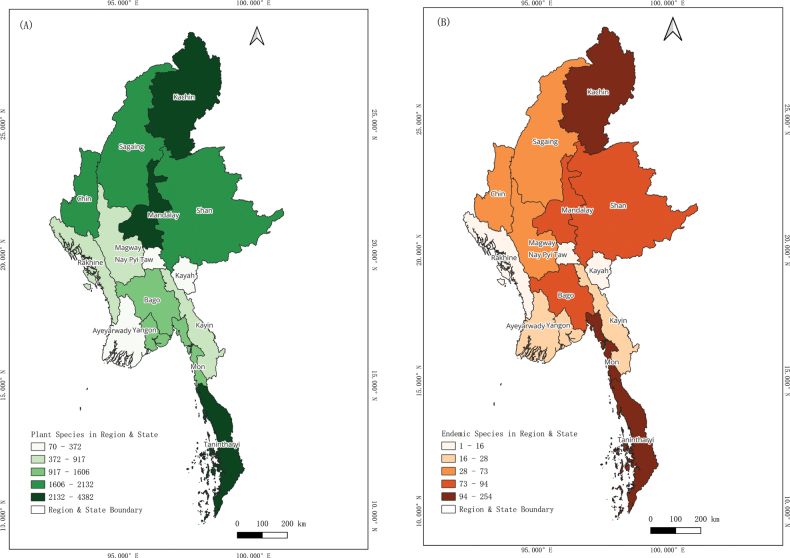
Plant Species Richness of Myanmar; A. the number of species in each Region and State of Myanmar, and B. the number of endemic species in each Region and State of Myanmar.

In comparison, the number of species was increasingly recorded in the periodically revised checklist editions including the present updated checklist (Fig. 3). Local distribution information was provided for almost all species, usually at Region and State level distribution ranges (Fig. 4A). Specific locality information was also provided for some species if known. The summary tables of families and genera are provided in the Suppl. materials 2, 3.

As for endemism, there are approximately 864 endemic species of vascular plants (6% of total species) in Myanmar. The distribution data of Myanmar endemic species is relatively coarse and currently possible at Region or State level, reflecting that the extensive collection efforts on these endemic species are obviously required for the effective conservation (Fig. 4B). Perhaps the narrowly distributed species will be at a high risk of extinction unless proper conservation actions are taken urgently.

**Figure 5.**
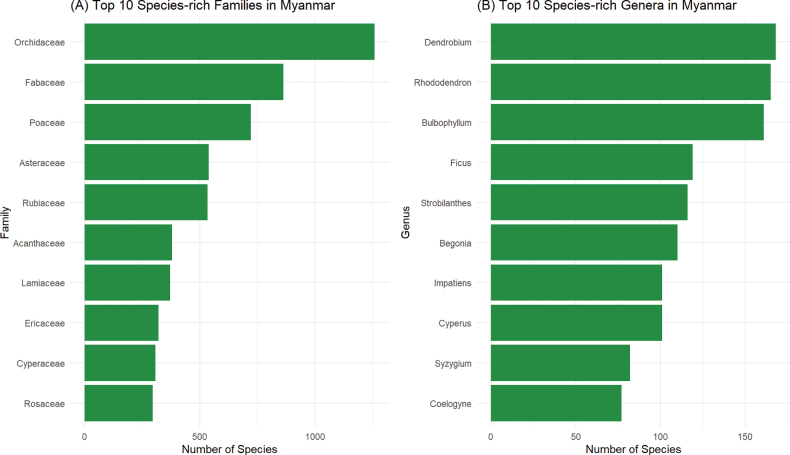
Species Richness of Myanmar flora. A. Top 10 species-rich families of vascular plants in Myanmar, and B. Top 10 species-rich genera of vascular plants in Myanmar.

As for species-rich families, the top 10 largest families of Myanmar flora, namely Orchidaceae, Fabaceae, Poaceae, Asteraceae, Rubiaceae, Acanthaceae, Lamiaceae, Ericaceae, Cyperaceae, Rosaceae, hold ca. 39.83% of total species (Fig. 5A). Among them, family Orchidaceae is exceptionally rich in the number of species, having more than 1,200 species. In addition, family Fabaceae, family Poaceae, family Asteraceae and family Rubiaceae hold more than 500 species in each.

As for species-rich genera, the top 10 largest genera of Myanmar flora, namely *Dendrobium*, *Rhododendron*, *Bulbophyllum*, *Ficus*, *Strobilanthes*, *Begonia*, *Impatiens*, *Cyperus*, *Syzygium*, *Coelogyne* hold ca. 8.56% of the total species (Fig. 5B). Among them, genus *Dendrobium*, genus *Rhododendron* and genus *Bulbophyllum* consist of more than 150 species in each.

### ﻿Floristic studies in Myanmar

The present study investigated the species richness of Myanmar flora mainly based on three main sources such as herbarium specimens, taxonomic literature and online databases. These three main sources contributed to the updated checklist dataset. It is noted that herbarium specimens are the backbone sources on which taxonomic literature and online databases were established.

In total, there were more than 50,000 herbarium specimens examined, including specimens of our own collections kept at both herbarium (PE) and herbarium (RAF), and specimens from the relevant online herbaria including Flora of Myanmar Database and Myanmar Vascular Plants Database. In the updated checklist, 6,194 species are directly sourced from herbarium specimens cited, whereas 4,673 species are sourced from literature and 3,153 species from online databases (GBIF & WCVP). It is noted that some species can be found in all three sources, while some species can be found in only one source. For species included in all three sources, the herbarium specimens are regarded as the preferred cited source rather than other two sources.

Based on specimens kept at various herbaria (including online herbaria), voucher specimen citations (ca. 18,236 specimens) are provided for 6,194 species. In cases where herbarium specimens are lacking, the species occurrences are mainly based on the most reliable references. It is evident that the botanical collection efforts are largely uneven across the different ecosystems of the country, reflecting that vast areas of ecosystems, particularly forest ecosystems, remain under-explored.

**Figure 6.**
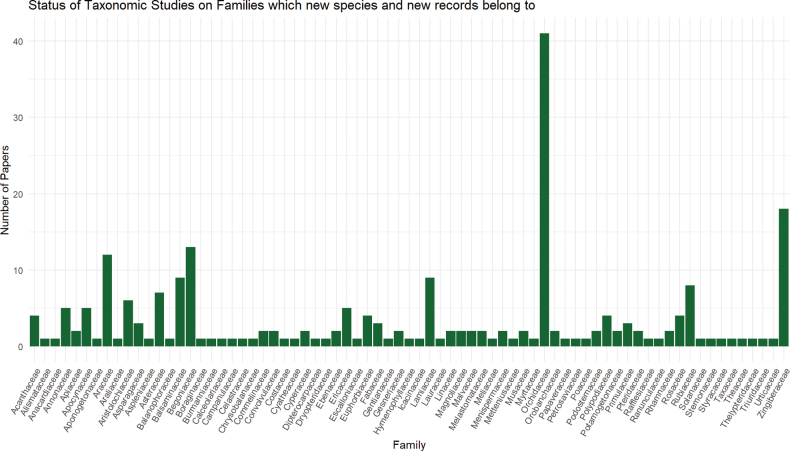
Status of Taxonomic studies on vascular plant families to which recently published new species and new records belong.

As for taxonomic literature, there were 168 taxonomic references on vascular plant species of Myanmar, from which the species information was extracted and verified in consultation with updated taxonomic classification systems and online databases. It is noted that the recently discovered new species and new records belong to 73 families of Myanmar flora (Fig. 6). The list of references for species data is provided in the Suppl. material 5.

### ﻿Taxonomic updates of Myanmar Flora

The familial circumscriptions as well as generic delimitations were largely updated due to many advances in plant taxonomy and systematics in recent years. Particularly, ca. 3,853 species, 314 genera and 56 families from the previous checklist (Kress et al. 2003) were subject to taxonomic changes due to the modern taxonomic classification systems. In this regard, the taxonomic updates on these families and genera of the previous checklist (Kress et al. 2003) are summarized and the first one is shown in Table 2 and the latter one shown in the Suppl. materials 4.

**Table 2.** Families of the previous checklist (Kress et al. 2003) which were subject to taxonomic changes according to the modern taxonomic classification systems.

No.	Old family	Accepted family	No.	Old family	Accepted family
1	Aceraceae	Sapindaceae	29	Hyacinthaceae	Asparagaceae
2	Agavaceae	Asparagaceae	30	Hydnoraceae	Aristolochiaceae
3	Alangiaceae	Cornaceae	31	Hydrophyllaceae	Boraginaceae
4	Alliaceae	Amaryllidaceae	32	Illiciaceae	Schisandraceae
5	Aloaceae	Asphodelaceae	33	Leeaceae	Vitaceae
6	Anthericaceae	Asparagaceae	34	Lemnaceae	Araceae
7	Aralidiaceae	Torricelliaceae	35	Limnocharitaceae	Alismataceae
8	Asclepiadaceae	Apocynaceae	36	Meliosmaceae	Sabiaceae
9	Aucubaceae	Garryaceae	37	Mimosaceae	Fabaceae
10	Avicenniaceae	Acanthaceae	38	Morinaceae	Caprifoliaceae
11	Balanitaceae	Zygophyllaceae	39	Myrsinaceae	Primulaceae
12	Bombacaceae	Malvaceae	40	Najadaceae	Hydrocharitaceae
13	Buddlejaceae	Scrophulariaceae	41	Parnassiaceae	Celastraceae
14	Caesalpiniaceae	Fabaceae	42	Phormiaceae	Asphodelaceae
15	Callitrichaceae	Plantaginaceae	43	Plagiopteraceae	Celastraceae
16	Cecropiaceae	Urticaceae	44	Podoaceae	Anacardiaceae
17	Chenopodiaceae	Amaranthaceae	45	Punicaceae	Lythraceae
18	Cochlospermaceae	Bixaceae	46	Sterculiaceae	Malvaceae
19	Convallariaceae	Asparagaceae	47	Taccaceae	Dioscoreaceae
20	Corylaceae	Betulaceae	48	Taxodiaceae	Cupressaceae
21	Datiscaceae	Tetramelaceae	49	Tetracentraceae	Trochodendraceae
22	Dipsacaceae	Caprifoliaceae	50	Tiliaceae	Malvaceae
23	Dracaenaceae	Asparagaceae	51	Trapaceae	Lythraceae
24	Epacridaceae	Ericaceae	52	Trilliaceae	Melanthiaceae
25	Flacourtiaceae	Salicaceae	53	Turneraceae	Passifloraceae
26	Hemerocallidaceae	Asphodelaceae	54	Valerianaceae	Caprifoliaceae
27	Hippocastanaceae	Sapindaceae	55	Viscaceae	Santalaceae
28	Hippuridaceae	Plantaginaceae	56	Zannichelliaceae	Potamogetonaceae

In recent years, the discovery of new species and new records has led to an increase in the number of species of Myanmar flora. At the same time, the updated taxonomic classification systems largely impact on the taxonomic status of the species of the previous checklist (Kress et al. 2003), resulting in taxonomic changes in familial, generic and species delimitations. In this regard, the present study updated the checklist of Kress et al. 2003 into a taxonomically updated checklist of Myanmar flora. As a result, the number of species of the updated checklist increased by 2,220 species compared to the previous checklist (Kress et al. 2003), mainly due to the discovery of new species and new records.

In brief, the species sources that contributed to the updated checklist can be classified into three categories, namely (1) Direct from previous checklist (45%): species which had no taxonomic changes and were directly transferred from the previous checklist (Kress et al. 2003); (2) New additions (36%): species which were newly added to the updated checklist, mainly sourced from herbarium specimens, taxonomic literature and online databases; and (3) Revised from the previous checklist (19%): species which were subject to taxonomic changes and revised taxonomically from the previous checklist (Kress et al. 2003).

### ﻿Common names of Myanmar flora

Common names were included in the updated checklist to broaden its usability across diverse audiences including scientists, students, decision-makers, resource managers, conservationists, and local communities. Not all species of Myanmar flora have common names or vernacular names, but most commercial timber species and crop species have common names. In particular, 16% of total species of Myanmar flora (ca. 2,183 species) have common names.

### ﻿Regional distribution of Myanmar flora

**Figure 7.**
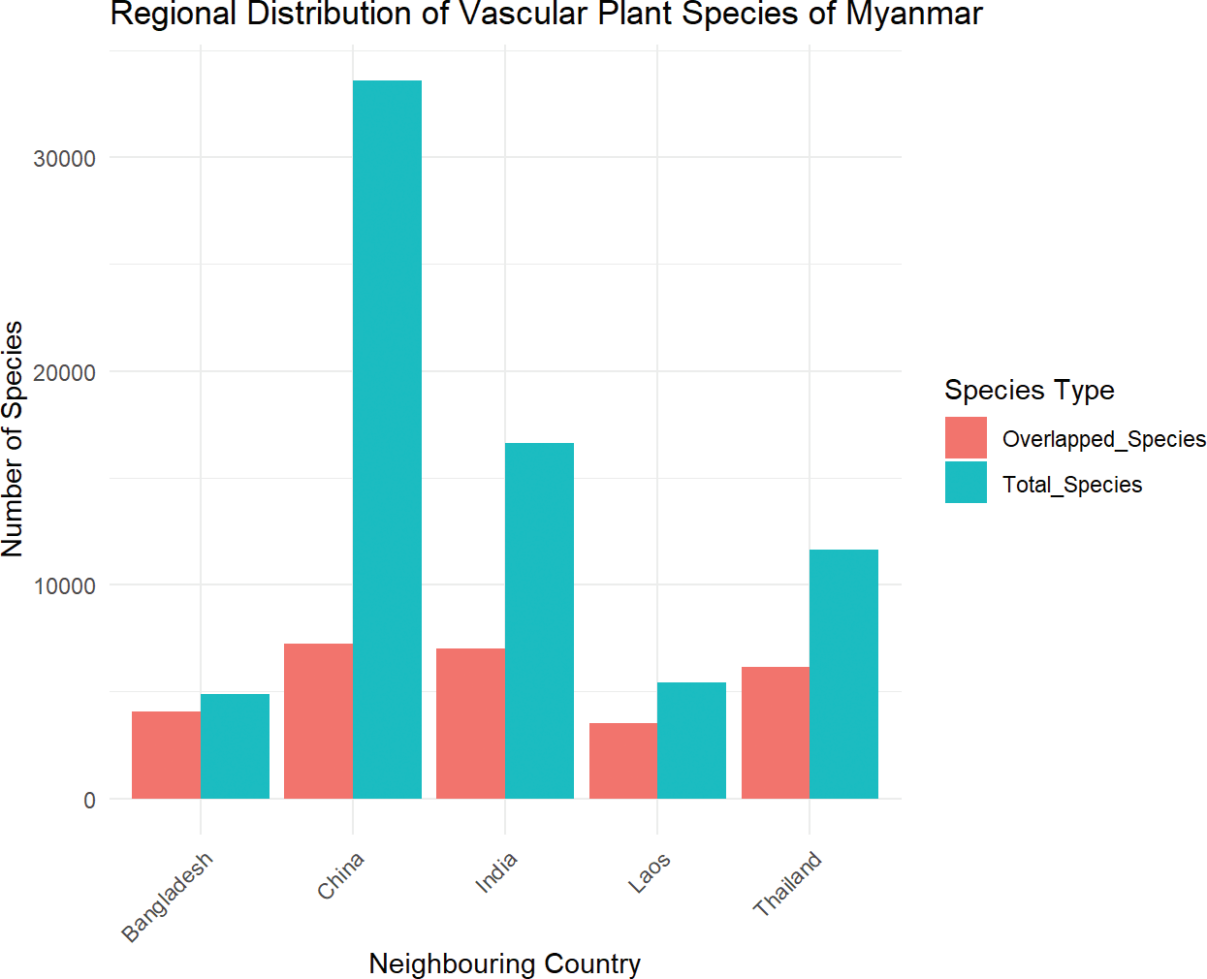
The regional distribution of vascular plant species of Myanmar, showing the number of plant species overlapped with the flora of the neighboring countries.

Regional distribution range of Myanmar vascular plants is usually broad on account of their extension into neighboring countries such as China, Laos, Thailand, India and Bangladesh. There are many species shared with the floristic diversity of the neighboring countries. For comparison, the plant species datasets for the neighboring countries were extracted from WCVP database by using rWCVP R package and cross checked with the updated checklist dataset for Myanmar flora so that the number of plant species that overlapped with the flora of the neighboring countries can be obtained, as shown in Fig. 7.

In sum, the updated checklist will serve as the taxonomically verified and specimen-based plant biodiversity data for the conservation planning and implementation in Myanmar although the updated checklist will need further revisions in the future.

### ﻿The updated checklist

The updated checklist consists of three major groups of vascular plant species of Myanmar; Angiosperms, Gymnosperms and Pteridophytes (see Suppl. material 1: appendix S1. Updated checklist of vascular plants of Myanmar).

The updated checklist consists of the following data:

1. Accepted names of families.
2. Accepted names of genera.
3. Accepted names of species, its author(s) and publication.
4. Distribution in Myanmar, usually Region and State (Provincial) level. In Myanmar, there are 15 administrative provinces including Nay Pyi Taw Union Territory, seven Regions (Ayeyarwaddy, Bago, Magway, Mandalay, Sagaing, Tanintharyi and Yangon) and seven States (Chin, Kachin, Kayah, Kayin, Mon, Rakhine and Shan). Specific localities are provided if known. The names of specific localities are mainly sourced from the cited herbarium specimens. All possible locality names are transcribed into clear modern versions, but some locality names are retained as verbatim names if not possible to transcribe or considered to keep as it is. In some cases, the locality information is too broad to get precise locations, particularly in some old collections. It is noted that the names and extent of a given locality (especially District levels) were periodically updated and changed over the past decades. For example, Bassein means Pathein, Moulmein means Mawlamyine, Rangoon means Yangon, Pegu means Bago and so on.
5. Specimen Citations: Collector (s), Collection number (s) and Herbarium codes (Index Herbariorum edited by Thiers BM, available at http://sweetgum.nybg.org/ih/). All specimens cited with an exclamation mark (!) have been examined. Some specimens without an exclamation mark (!) are studied and known from literature of new species description, new species records and species checklist.
6. Common Names (if any). Not all species had common names, but all possible common names were assigned to the respective species. It is noted that some common names belong to more than one species, vice versa.
7. Major Groups: Angiosperms, Gymnosperms, Pteridophytes.
8. Endemic Status: TRUE means endemic species of Myanmar while FALSE means not the endemic species of Myanmar.

## ﻿Discussion

### ﻿Plant species richness of Myanmar in a regional and global context

As one of the research milestones in floristic studies of Myanmar, the present study resulted in an updated checklist of the Myanmar flora, which includes 14,020 species in 2,701 genera and 292 families of vascular plants known from Myanmar, an increase of 19% more species than previously recorded. However, increased botanical investigations are still needed to better understand the plant biodiversity of the country.

Compared with neighboring countries with intensive floristic studies, the flora of Myanmar still lags for comprehensive documentation. For example, flora of China comprises ca. 38,520 species of vascular plants (including infraspecific taxa). With the ongoing botanical explorations by Chinese botanists, many new species are still increasingly discovered from China. Particularly, Yunnan Province of China holds ca. 16,772 species of vascular plants (including infraspecific taxa), sharing the border line area with Myanmar (Xie et al. 2021). In addition, it is estimated that there are more than 11,000 species of vascular plants in Thailand, one of the neighboring countries of Myanmar. Interestingly, the modern taxonomic treatments have been completed for about 60% of the estimated number of vascular plant species in Thailand, marking steady progress in its floristic studies (Middleton et al. 2019).

Globally, 350,386 taxonomically accepted species of vascular plants are distributed across the different ecosystems of the world (Antonelli et al. 2023). With advances in scientific exploration in various ecosystems and modern taxonomic revisions, discoveries of species new to science, occur at the rate of about 2,500 plant species per year (Antonelli et al. 2023).

Encompassing four major global biodiversity hotspots, Southeast Asia is a region of high richness of plant biodiversity, with increasing discoveries of many new species in recent years. It is estimated that approximately 50,000 flowering plant species are to be found in Southeast Asia (Middleton et al. 2019). In this regard, much more botanical exploration is needed to better understand the richness of plant biodiversity because most areas of the region still remain underexplored and some endemic plant species are narrowly distributed, i.e., only known from its type localities (Middleton et al. 2019).

The same situation is true in Myanmar. It is evident that the botanical collections are still needed to cover the whole floristic diversity of Myanmar because botanical explorations had sharply decreased in Myanmar since 1950, resulting in a large gap of knowledge on Myanmar flora (Kress et al. 2003). A recent study highlighted that the floristic collection density varies across different Regions and States of the country, showing the highest collection densities in Mandalay Region, Chin State and Yangon Region (Aung et al. 2023). It is noted that this possible collection bias should be considered in future botanical surveys in Myanmar.

Having vast areas of ecosystems of the country underexplored, many species are waiting to be discovered in Myanmar. Moreover, the baseline data on the number of plant species and its distribution range is required for effective plant conservation in Myanmar. In addition, modern taxonomic treatments for each family or genus are a pre-requisite for the better integration of updated taxonomic data into biodiversity conservation planning and implementation in Myanmar.

### ﻿Progress in floristic studies in Myanmar

As for plant biodiversity research, there are many achievements obtained from the cooperation between Myanmar Forest Department and various international institutions, such as the Chinese Academy of Sciences, Makino Botanical Garden, The National Museum of Nature and Science (Tokyo), Korean National Institute of Biological Resources, New York Botanical Garden, the Smithsonian Institution, Marburg University and Singapore Botanic Gardens in the recent years, resulting in discoveries of many new species as well as new records from Myanmar (Kurzweil and Lwin 2014; Khine et al. 2016; Aung et al. 2017, 2018, 2020, 2021; Aung and Jin 2018, 2021; Ding et al. 2018; Jin and Mint 2018; Jin et al. 2018; Yang et al. 2018; Hughes et al. 2019; Middleton et al. 2019; Ormerod et al. 2021; Fujiwara et al. 2022; Tanaka et al. 2022a; Tong et al. 2022; Wood et al. 2022; Floden et al. 2023; Jung et al. 2024).

In addition, some local researchers are also working on the taxonomic studies on flora of Myanmar in cooperation with their counterparts from international institutions, resulting in discoveries of new species as well as new records from Myanmar (Hein and Naive 2021; Hein et al. 2021; Naive and Hein 2021; Hein et al. 2022; Naive et al. 2022a, 2022b, 2024; Paing et al. 2024).

### ﻿Conservation efforts in Myanmar

As for plant biodiversity conservation, all species of fauna and flora are included under the national biodiversity conservation program, usually *in situ* conservation approach such as establishment of Protected Areas across the country. In Myanmar, there are 61 protected areas (6.43% of the country’s total area) already established across different ecosystems of the country, including tropical and subtropical mountain forest ecosystems, wetland ecosystems (seven Ramsar sites), coastal and marine ecosystems and so on (FD 2020). In addition, 10-year program of Re-establishing Natural Habitats (2019–2029) has been implemented in 19 targeted protected areas across Myanmar in order to restore the natural habitats of various species of fauna and flora (FD 2019).

**Figure 8.**
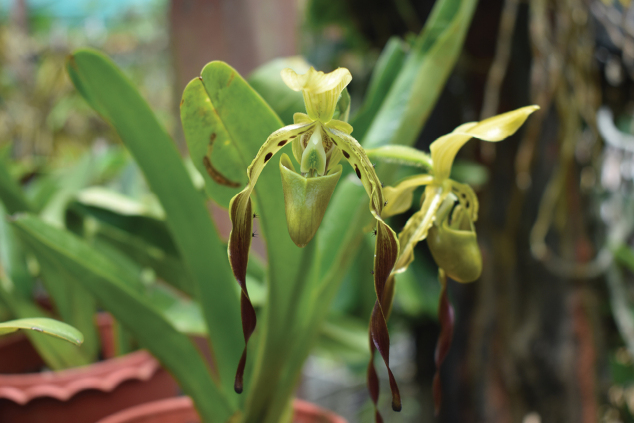
*Paphiopedilumparishii* (Rchb.) Pfitzer which is proposed as a flagship species for biodiversity conservation in Myanmar. Photo by Ye Lwin Aung.

In terms of *ex situ* conservation approach, it is noted that the seeds of ca. 400 orchid species from Myanmar have been deposited at the Svalbard Global Seed Vault, Norway, for the purpose of *ex situ* conservation measures on Myanmar orchid flora since 2018 (Svalbard Global Seed Vault 2018). Most orchid species, particularly *Paphiopedilum* species, are very rare in the wild and endangered under various threats so that all orchid species are enlisted on CITES appendices. In this regard, *Paphiopedilumparishii* (Rchb.) Pfitzer is proposed here as a flagship species for biodiversity conservation in Myanmar because this species is native to Myanmar and its conservation status is currently assigned as Endangered (EN) in the IUCN Red List of Threatened Species (Rankou and Averyanov 2015) (Fig. 8).

As for legal protection, 52 tree species of commercial or conservation importance, all species of family Orchidaceae and 14 herbaceous plant species of conservation concerns, are legally protected by national legislation under the authority of Myanmar Forest Department (FD 2020).

### ﻿Challenges for biodiversity research and conservation

There are many challenges in the implementation of plant biodiversity research and conservation in Myanmar, for example, habitat loss or fragmentation due to land use change and deforestation, climate change impacts, demographic dynamics (e.g., population growth trends), political instability and so on. It is evident that local communities depend on natural resources for their livelihoods, e.g., food and income generated by collection of forest products (e.g., bamboos or mushrooms) from natural forest land (Chew et al. 2023; Mon et al. 2023; Toda et al. 2023). Under such circumstances, the sustainability of biodiversity resources will largely support the sustainability of local communities’ livelihoods. In this regard, community-based as well as nature-based solutions will secure the sustainable development of the country in the long run.

The role of citizen science has become popular and significant in community-based biodiversity research and conservation in the world. The same is also true in Myanmar, e.g., the recent discovery of a new plant species from Myanmar, *Begoniakayinensis* M.B.Maw & Y.H.Tan discovered from Kayin State of Myanmar with the citizen science-stimulated attempts (Maw et al. 2023).

In the State of the World’s Plants and Fungi 2023, the factors affecting the species description rate were highlighted as follows; (1) conflict and instability, (2) type of terrain and access, (3) economic barriers, (4) number of trained taxonomists, and (5) access to reference collections and data (Antonelli et al. 2023). Like most tropical biodiversity-rich developing countries, Myanmar also needs to deal with these factors for its biodiversity research and conservation actions. For example, there are some new species of Myanmar flora recently described from old collections of herbarium specimens, namely, *Pinaliataunggyiensis* Ormerod & Kurzweil described in 2020 based on the herbarium specimen (F. G. Dickason 8282) collected on 26 April 1939, and *Pinaliashiuyingiana* Ormerod & E.W.Wood described in 2010 based on the herbarium specimen (F. Kingdon Ward 20970) collected on 9 June 1953, both specimens kept at herbarium (AMES) (Ormerod and Wood 2010; Ormerod and Kurzweil 2020). These examples reflect that there is a significant gap between the year of collection and the year of formal description, with many decades elapsing before the taxonomic identification and description of these new species from Myanmar are completed.

Among the key challenges for plant biodiversity research and conservation in Myanmar, political instability is the most prominent challenge to deal with as soon as possible because it even affects other challenges and issues such as deforestation, climate change, economic growth, population growth, and so on. Its impacts are unpredictably large on biodiversity research and conservation in Myanmar. The efforts to reach a sound political resolution are of considerable significance not only for the well-being of the nation and its people but also for the effective conservation of biodiversity.

### ﻿Limitations of present study

There are two key limitations in the present study, namely (1) limited availability of herbarium specimens, and (2) limited taxonomic studies on Myanmar flora. Only 44% of total species have specimen citations, while the remaining ones are sourced from literature and online databases. Obviously, intensive botanical collections are needed to fill such gaps of specimen requirements. Fortunately, the online herbaria provided access to herbarium specimen records. Among these digital specimens, some are assigned with species level identification while some are assigned with genus or family level identification, requiring specimen examinations by plant taxonomists. Currently, the species without specimen citations are assigned with literature citations or respective online database citations. In the future, the botanical collections are expected to fill the large gaps of specimen requirements.

Another key issue is the limited taxonomic studies on Myanmar flora, evidenced by limited studies on ferns and lycophytes as well as bryophytes of Myanmar. Perhaps there were very few plant taxonomists in Myanmar and the international collaborations were also very few in past decades. Currently, all available taxonomic literature were consulted to include all relevant taxonomic work in the updated checklist.

### ﻿Recommendations for future directions

Therefore, the following recommendations are proposed for development of plant biodiversity research and conservation in Myanmar; (1) scaling up the development of research infrastructure and capacity building of research personnel working in various fields of life science, (2) strengthening the collaboration and cooperation with international biodiversity research and conservation organizations, (3) seeking funding opportunities from various funding organizations such as central government, conservation and research foundations, international research organizations and so on, (4) raising the public awareness on biodiversity conservation by means of organizing the citizen science-stimulated conservation activities, the conservation education programs at schools and universities as well as public parks, and (5) encouraging the local researchers or taxonomists to assign the new species names with their formal scientific names as well as their vernacular names or local names (if any or created on the author’s own) in order to make the local communities familiar with the new species and to raise its respective conservation awareness among the societies.

## ﻿Additional information

### Use of AI

No use of AI was reported.

### Author ORCIDs

Ye Lwin Aung https://orcid.org/0000-0002-6765-598X

Yunhong Tan https://orcid.org/0000-0001-6238-2743

### Data availability

All of the data that support the findings of this study are available in the main text or Supplementary Information.

## Supplementary materials

Supplementary material 1 Appendix S1: Updated checklist of vascular plants of Myanmar Data type xlsx Explanation note An updated checklist consists of 14,020 species in 2,701 genera and 292 families of vascular plants known from Myanmar. Among them, there are 13,314 native species and 706 introduced species in Myanmar. In Myanmar, there are 864 endemic species which need proper conservation actions. https://binary.pensoft.net/file/1392451This dataset is made available under the Open Database License (http://opendatacommons.org/licenses/odbl/1.0/). The Open Database License (ODbL) is a license agreement intended to allow users to freely share, modify, and use this Dataset while maintaining this same freedom for others, provided that the original source and author(s) are credited.Ye Lwin Aung, Mung Htoi Aung, Yunhong Tan, Xiaohua Jin

Supplementary material 2 Summary table of families with their respective number of genera and species in the updated checklist Data type xlsx https://binary.pensoft.net/file/1392452This dataset is made available under the Open Database License (http://opendatacommons.org/licenses/odbl/1.0/). The Open Database License (ODbL) is a license agreement intended to allow users to freely share, modify, and use this Dataset while maintaining this same freedom for others, provided that the original source and author(s) are credited.Ye Lwin Aung, Mung Htoi Aung, Yunhong Tan, Xiaohua Jin

Supplementary material 3 Summary table of genera with their respective number of species in updated checklist Data type xlsx https://binary.pensoft.net/file/1392453This dataset is made available under the Open Database License (http://opendatacommons.org/licenses/odbl/1.0/). The Open Database License (ODbL) is a license agreement intended to allow users to freely share, modify, and use this Dataset while maintaining this same freedom for others, provided that the original source and author(s) are credited.Ye Lwin Aung, Mung Htoi Aung, Yunhong Tan, Xiaohua Jin

Supplementary material 4 Genera of the previous checklist (Kress et al. 2003) Data type pdf Explanation note Genera of the previous checklist (Kress et al. 2003) which were subject to taxonomic changes according to the modern taxonomic classification systems. https://binary.pensoft.net/file/1392454This dataset is made available under the Open Database License (http://opendatacommons.org/licenses/odbl/1.0/). The Open Database License (ODbL) is a license agreement intended to allow users to freely share, modify, and use this Dataset while maintaining this same freedom for others, provided that the original source and author(s) are credited.Ye Lwin Aung, Mung Htoi Aung, Yunhong Tan, Xiaohua Jin

Supplementary material 5 List of references for Myanmar plant species data Data type pdf https://binary.pensoft.net/file/1392455This dataset is made available under the Open Database License (http://opendatacommons.org/licenses/odbl/1.0/). The Open Database License (ODbL) is a license agreement intended to allow users to freely share, modify, and use this Dataset while maintaining this same freedom for others, provided that the original source and author(s) are credited.Ye Lwin Aung, Mung Htoi Aung, Yunhong Tan, Xiaohua Jin
