# Supplementary material for: ﻿An updated checklist of vascular plants of Myanmar
Source: PhytoKeys. 2025 Aug 11;261:135–64. doi: 10.3897/phytokeys.261.154986 (PMC12361923; doi:10.3897/phytokeys.261.154986)
Supplement: Supplementary material 4 — Genera of the previous checklist (Kress et al. 2003) [file phytokeys-261-135_article-154986__-s004.pdf]

Table 1. Genera of the previous checklist (Kress et al. 2003) which were subject to taxonomic changes according to the modern taxonomic classification systems

No.	Old genus	Accepted genus	Remark
1	*Abarema*	*Archidendron*	
2	*Aberia*	*not traceable in global authoritative databases such as POWO, WFO, IPNI*	Note1. One species recorded from Myanmar, namely *Aberia gardnerii* Clos is missing in the global authoritative databases such as POWO, WFO and IPNI. Therefore, the genus and its species are currently set aside from the present checklist
3	*Acanthephippium*	*Acanthophippium*	
4	*Achras*	*Manilkara*	
5	*Acmena*	*Syzygium*	
6	*Acrocephalus*	*Platostoma*	
7	*Adelostemma*	*Cynanchum*	
8	*Adenosacme*	*Mycetia*	
9	*Adhatoda*	*Justicia*	
10	*Aechmanthera*	*Strobilanthes*	
11	*Agonosma*	*Aganosma*	
12	*Alaeanthus*	*Allaeanthus*	
13	*Allomorphia*	*Oxyspora*	
14	*Althaea*	*Alcea*	
15	*Altingia*	*Liquidambar*	
16	*Alyssum*	*Ptilotrichum*	
17	*Amitostigma*	*Hemipilia*	
18	*Anagallis*	*Lysimachia*	
19	*Andrachne*	*Leptopus*	
20	*Anogeissus*	*Terminalia*	
21	*Anotis*	*Neanotis*	
22	*Anplectrum*	*Diplectria*	
23	*Antenoron*	*Persicaria*	
24	*Anthocephalus*	*Neolamarckia*	
25	*Aporusa*	*Aporosa*	
26	*Apteron*	*Ventilago*	
27	*Ascocentrum*	*Vanda*	
28	*Atherolepis*	*Finlaysonia*	
29	*Atherostemon*	*Atherandra*	
30	*Atylosia*	*Cajanus*	

No.	Old genus	Accepted genus	Remark
31	*Aulacodiscus*	*Urophyllum*	
32	*Balanostreblus*	*Streblus*	
33	*Belamcanda*	*Iris*	
34	*Bennettia*	*Bennettiodendron*	
35	*Benthamidia*	*Cornus*	
36	*Borreria*	*Spermacoce*	
37	*Boscia*	*Hypselandra*	
38	*Boussingaultia*	*Anredera*	
39	*Brachiaria*	*Urochloa*	
40	*Brachycome*	*Brachyscome*	
41	*Brachystelma*	*Ceropegia*	
42	*Brassaia*	*Heptapleurum*	
43	*Calamintha*	*Clinopodium*	
44	*Callistemon*	*Melaleuca*	
45	*Campanumoea*	*Cyclocodon*	
46	*Cardanthera*	*Hygrophila*	
47	*Carinta*	*Geophila*	
48	*Caryopteris*	*Pseudocaryopteris*	
49	*Caucalis*	*Torilis*	
50	*Caulokaempfera*	*Monolophus*	
51	*Cedrela*	*Toona*	
52	*Celsia*	*Verbascum*	
53	*Cephaelis*	*Psychotria*	
54	*Cephalandra*	*Coccinia*	
55	*Cephalantheropsis*	*Calanthe*	
56	*Cephalocroton*	*Cladogynos*	
57	*Cephalostigma*	*Wahlenbergia*	
58	*Chaetoseris*	*Melanoseris*	
59	*Chamaecladon*	*Homalomena*	
60	*Chamerion*	*Epilobium*	
61	*Chionachne*	*Polytoca*	
62	*Chirita*	*Henckelia*	
63	*Chusua*	*Hemipilia*	
64	*Cimicifuga*	*Actaea*	
65	*Cleistocalyx*	*Syzygium*	
66	*Cnicus*	*Cirsium*	
67	*Coccoceras*	*Mallotus*	
68	*Coelodiscus*	*Mallotus*	
69	*Corallobotrys*	*Agapetes*	
70	*Costus*	*Hellenia*	
71	*Cotylanthera*	*Exacum*	
72	*Cudrania*	*Maclura*	

No.	Old genus	Accepted genus	Remark
73	*Curcumorpha*	*Boesenbergia*	
74	*Cyclostemon*	*Drypetes*	
75	*Cydista*	*Bignonia*	
76	*Cydonia*	*Chaenomeles*	
77	*Cylista*	*Paracalyx*	
78	*Cyperorchis*	*Cymbidium*	
79	*Cystacanthus*	*Phlogacanthus*	
80	*Daedalacanthus*	*Eranthemum*	
81	*Daemonorops*	*Calamus*	
82	*Delima*	*Tetracera*	
83	*Dendrocalamopsis*	*Bambusa*	
84	*Dendrochilum*	*Coelogyne*	
85	*Desmostachys*	*Desmostachya*	
86	*Dianthera*	*Justicia*	
87	*Dicentra*	*Dactylicapnos*	
88	*Dichroa*	*Hydrangea*	
89	*Dilochia*	*Thuniopsis*	Note 2. Although the genus name is taxonomically accepted, its species Dilochia subsessilis (Rolfe) S. Thomas is synonym of Thuniopsis subsessilis (Rolfe) Ormerod, Kurzweil & Schuit. according to WFO.
90	*Dioclea*	*Macropsychanthus*	
91	*Diplarche*	*Rhododendron*	
92	*Diplomeria*	*Diplomeris*	
93	*Diplycosia*	*Gaultheria*	
94	*Dipteracanthus*	*Ruellia*	
95	*Distemon*	*Neodistemon*	
96	*Dregea*	*Wattakaka*	
97	*Drymoda*	*Bulbophyllum*	
98	*Dufrenoya*	*Dendrotrophe*	
99	*Dysophylla*	*Pogostemon*	
100	*Ebermaiera*	*Staurogyne*	
101	*Echites*	*Echites*	Note 3. Although the genus is taxonomically accepted, its two species recorded from Myanmar, namely *Echites glandulifera* Wall. & *Echites odoratissima* Griff. are not traceable to check their

No.	Old genus	Accepted genus	Remark
			taxonomic status because these two species names are missing in the globally authoritative databases such as POWO, WFO and IPNI. Therefore, the genus and its two species are currently set aside from the present checklist.
102	*Eichhornia*	*Pontederia*	
103	*Elettariopsis*	*Amomum*	
104	*Ellipeia*	*Trivalvaria*	
105	*Ellipeiopsis*	*Uvaria*	
106	*Emblica*	*Phyllanthus*	
107	*Emmenopterys*	*Schizomussaenda*	
108	*Engelhardtia*	*Engelhardia*	
109	*Enhydra*	*Enydra*	
110	*Enicosanthum*	*Monoon*	
111	*Epigeneium*	*Dendrobium*	
112	*Eremopogon*	*Schizachyrium*	
113	*Erianthus*	*Narenga*	
114	*Eriolobus*	*Eriolobus*	Note 4. Although the genus is taxonomically accepted, its two species recorded from Myanmar, namely Eriolobus hookeriana Decne. & Eriolobus indica Schneid. are not traceable to check their taxonomic status because these two species names are missing in the globally authoritative databases such as POWO, WFO and IPNI. Therefore, the genus and its two species are currently set aside from the present checklist.
115	*Esmeralda*	*Arachnis*	
116	*Eucharis*	*Urceolina*	Note 5. cultivated but lacking occurrence evidence for Myanmar
117	*Eurycles*	*Proiphys*	

No.	Old genus	Accepted genus	Remark
118	*Fioria*	*Hibiscus*	
119	*Flickingeria*	*Dendrobium*	
120	*Gaertnera*	*Psychotria*	
121	*Gelonium*	*Suregada*	
122	*Geniosporum*	*Platostoma*	
123	*Geodorum*	*Eulophia*	
124	*Gonatanthus*	*Remusatia*	
125	*Gonioscypha*	*Rohdea*	
126	*Gordonia*	*Polyspora*	
127	*Gunnarorchis*	*Thelasis*	
128	*Gymnopetalum*	*Trichosanthes*	
129	*Gynandropsis*	*Cleome*	
130	*Gynopogon*	*Alyxia*	
131	*Haloragis*	*Haloragis*	Note 6. Although the genus is taxonomically accepted, its one species recorded from Myanmar, namely Haloragis philipinensis Merr. is not traceable to check their taxonomic status because that one species name is missing in the globally authoritative databases such as POWO, WFO and IPNI. Therefore, the genus and its one species are currently set aside from the present checklist.
132	*Helicodiceras*	*Helicodiceros*	
133	*Helictotrichon*	*Tzveleviochloa*	
134	*Helipterum*	*Rhodanthe*	
135	*Hemicyclia*	*Drypetes*	
136	*Hemistrepta*	*not traceable in global authoritative databases such as POWO, WFO, IPNI*	Note 7. This genus name and its one species recorded from Myanmar, namely Hemistrepta lyrata Bunge are missing in global authoritative databases such as POWO, WFO, IPNI. Therefore, these names are currently set aside from the present checklist.
137	*Hemsleya*	*Gomphogyne*	
138	*Hesperethusa*	*Naringi*	

No.	Old genus	Accepted genus	Remark
139	*Hippocratea*	*Reissantia*	
140	*Hitchenia*	*Curcuma*	
141	*Holboellia*	*Stauntonia*	
142	*Holostemma*	*Cynanchum*	
143	*Hydnora*	*Hydnora*	Note 8. Although the genus is taxonomically accepted, its one species recorded from Myanmar, namely Hydnora angustifolia Roxb. is not traceable to check their taxonomic status because that one species name is missing in the globally authoritative databases such as POWO, WFO and IPNI. Therefore, the genus and its one species are currently set aside from the present checklist.
144	*Hygrochilus*	*Phalaenopsis*	
145	*Hymenopogon*	*Neohymenopogon*	
146	*Hyphear*	*Taxillus*	
147	*Ilysanthes*	*Lindernia*	
148	*Indochloa*	*Euclasta*	
149	*Indopolysolenia*	*Leptomischus*	
150	*Ione*	*Bulbophyllum*	
151	*Ismelia*	*Glebionis*	
152	*Isonandra*	*Palaquium*	
153	*Isopyrum*	*Dichocarpum*	
154	*Isotoma*	*Hippobroma*	Note 9. introduced species, but lacking occurrence evidence for Myanmar
155	*Jurinea*	*Dolomiaea*	
156	*Kalimpongia*	*Dickasonia*	
157	*Kickxia*	*Nanorrhinum*	
158	*Kirganelia*	*Phyllanthus*	
159	*Klemachloa*	*Dendrocalamus*	
160	*Kochia*	*Bassia*	
161	*Kurrimia*	*Bhesa*	
162	*Kyllinga*	*Cyperus*	
163	*Lansium*	*Sphaerosacme*	
164	*Lens*	*Vicia*	
165	*Lettsomia*	*Blinkworthia*	

No.	Old genus	Accepted genus	Remark
166	*Libocedrus*	*Calocedrus*	
167	*Linaria*	*Nanorrhinum*	
168	*Lipocarpha*	*Cyperus*	
169	*Listera*	*Neottia*	
170	*Lloydia*	*Gagea*	
171	*Lophophyllum*	*Cyclea*	
172	*Lourea*	*Uraria*	
173	*Lychnis*	*Silene*	Note 10. one species recorded from Myanmar, namely Lychnis inflata Wall. is synonym of Silene setaesperma Majumdar according to POWO.
174	*Lycopersicon*	*Solanum*	
175	*Maba*	*Diospyros*	
176	*Macfadyena*	*Dolichandra*	
177	*Mahonia*	*Berberis*	
178	*Manglietia*	*Magnolia*	
179	*Manisuris*	*Hemarthria*	
180	*Mantisia*	*Globba*	
181	*Maoutia*	*Leucosyke*	
182	*Mathiola*	*Mathiola W.T.Aiton, Hort. Kew., ed. 2 [W.T. Aiton] 4: 119 (1812), nom. inval.*	Note 11. invalid name
183	*Melanorrhoea*	*Gluta*	
184	*Melanthera*	*Wollastonia*	
185	*Melodorum*	*Goniothalamus*	
186	*Melothria*	*Solena & Cucumis*	Note 12. Although the genus Melothria is taxonomically accepted, its two species recorded from Myanmar, namely Melothria heterophylla (Lour.) Cogn. & Melothria maderaspatana (L.) Cogn. are synonyms of Solena heterophylla Lour. & Cucumis maderaspatanus L. respectively.
187	*Menispermum*	*Menispermum*	Note 13. This genus name is taxonomically accepted but its one species recorded from Myanmar, namely

No.	Old genus	Accepted genus	Remark
			Menispermum dioicum Griff. is currently assigned as unplaced name according to POWO. Therefore, the genus and its one species are currently set aside from the present checklist.
188	*Metadina*	*Adina*	
189	*Michelia*	*Magnolia*	
190	*Mischobulbum*	*Tainia*	
191	*Modecca*	*Adenia*	
192	*Moghania*	*Flemingia*	
193	*Molineria*	*Curculigo*	
194	*Monochoria*	*Pontederia*	
195	*Monomeria*	*Bulbophyllum*	
196	*Moschosma*	*Basilicum*	
197	*Mukia*	*Cucumis*	
198	*Myrioneuron*	*Mycetia*	
199	*Naravelia*	*Clematis*	
200	*Neohouzeaua*	*Schizostachyum*	
201	*Neotrigonostemon*	*Trigonostemon*	
202	*Nepenthandra*	*Trigonostemon*	
203	*Niebuhria*	*Maerua*	
204	*Nomaphila*	*Hygrophila*	
205	*Nomocharis*	*Lilium*	
206	*Nothochaeta*	*Phlomoides*	
207	*Notonia*	*Caputia*	
208	*Ochrocarpos*	*Mammea*	
209	*Octotropis*	*Prismatomeris*	
210	*Oleoxylon*	*Dipterocarpus*	
211	*Ophiorrhiziphyllon*	*Staurogyne*	
212	*Oritrephes*	*Pseudodissochaeta*	
213	*Ornithochilus*	*Phalaenopsis*	
214	*Otanthera*	*Melastoma*	
215	*Oxymitra*	*Friesodielsia*	
216	*Oxytenanthera*	*Gigantochloa*	
217	*Paracarvum*	*Hackelia*	
218	*Parameria*	*Urceola*	
219	*Parvatia*	*Stauntonia*	
220	*Pedilanthus*	*Euphorbia*	
221	*Pegaeophyton*	*Aphragmus*	
222	*Pennisetum*	*Cenchrus*	

No.	Old genus	Accepted genus	Remark
223	*Pentanura*	*Decalepis*	
224	*Pentapanax*	*Aralia*	
225	*Pentapterygium*	*Agapetes*	
226	*Petraea*	*Petrea*	
227	*Phacelurus*	*Thyrsia*	
228	*Phlomis*	*Phlomoides*	
229	*Phylloboea*	*Paraboea*	
230	*Phyllodesmis*	*Taxillus*	
231	*Physostelma*	*Hoya*	
232	*Pimelandra*	*Sadiria*	
233	*Pisum*	*Lathyrus*	
234	*Plecospermum*	*Maclura*	
235	*Plectronia*	*Eleutherococcus*	
236	*Pleurospermum*	*Hymenidium*	
237	*Podostemum*	*Polypleurum*	
238	*Polianthes*	*Agave*	
239	*Pommereschia*	*Pommereschea*	
240	*Ponerorchis*	*Sirindhornia*	
241	*Pouteria*	*Planchonella*	
242	*Pratia*	*Lobelia*	
243	*Priotropis*	*Crotalaria*	
244	*Pseudostreblus*	*Streblus*	
245	*Pterostyrax*	*Rehderodendron*	
246	*Quamoclit*	*Ipomoea*	
247	*Quisqualis*	*Combretum*	
248	*Rapanea*	*Myrsine*	
249	*Raphistemma*	*Cynanchum*	
250	*Remirea*	*Cyperus*	
251	*Rhodeleia*	*Rhodoleia*	
252	*Rhynchelytrum*	*Melinis*	
253	*Ripidium*	*Tripidium*	
254	*Roydsia*	*Stixis*	
255	*Ryparia*	*Ryparosa*	
256	*Sansevieria*	*Dracaena*	
257	*Sarcostemma*	*Cynanchum*	
258	*Saritaea*	*Bignonia*	
259	*Sauropus*	*Breynia*	
260	*Schefflera*	*Heptapleurum*	
261	*Schismatoglottis*	*Apoballis*	
262	*Schizophragma*	*Hydrangea*	
263	*Scyphopetalum*	*Paranephelium*	
264	*Sebastiania*	*Microstachys*	

No.	Old genus	Accepted genus	Remark
265	*Sechium*	*Sicyos*	
266	*Securinega*	*Flueggea*	
267	*Selinum*	*Ligusticum*	
268	*Seseli*	*Psammogeton*	
269	*Siegesbeckia*	*Sigesbeckia*	
270	*Sisymbrium*	*Alliaria*	
271	*Smilacina*	*Maianthemum*	
272	*Smithatris*	*Curcuma*	
273	*Souliea*	*Actaea*	
274	*Spathichlamys*	*Greenea*	
275	*Spiradiclis*	*Ophiorrhiza*	
276	*Stahlianthus*	*Curcuma*	
277	*Statice*	*Limonium*	
278	*Staurochilus*	*Trichoglottis*	
279	*Stauropsis*	*Vandopsis*	
280	*Stemodia*	*Limnophila*	
281	*Stenoseris*	*Melanoseris*	
282	*Streptocarpus*	*Damrongia*	
283	*Sumbavia*	*Sumbaviopsis*	
284	*Sunipia*	*Bulbophyllum*	
285	*Syncalathium*	*Melanoseris*	
286	*Taeniochlaena*	*Rourea*	
287	*Talipariti*	*Hibiscus*	
288	*Tapirira*	*Pegia*	
289	*Taxotrophis*	*Streblus*	
290	*Teinostachyum*	*Schizostachyum*	
291	*Thevetia*	*Cascabela*	
292	*Thryallis*	*Galphimia*	
293	*Touchiroa*	*Crudia*	
294	*Trachylobium*	*Hymenaea*	
295	*Tractocopevodia*	*Melicope*	
296	*Treisteria*	*Torenia*	
297	*Trias*	*Bulbophyllum*	
298	*Trichurus*	*Trichuriella*	
299	*Triraphis*	*Neyraudia*	
300	*Tristania*	*Tristaniopsis*	
301	*Tupidanthus*	*Heptapleurum*	
302	*Turraea*	*Munronia*	
303	*Tutcheria*	*Pyrenaria*	
304	*Tylophora*	*Vincetoxicum*	
305	*Unona*	*Cananga*	
306	*Urginea*	*Drimia*	

No.	Old genus	Accepted genus	Remark
307	*Ventricularia*	*Trichoglottis*	
308	*Vinca*	*Catharanthus*	
309	*Wagatea*	*Moullava*	
310	*Webera*	*Tarennoidea*	
311	*Wormia*	*Dillenia*	
312	*Xanthosoma*	*Phyllotaenium*	Note 14. cultivated but lacking occurrence evidence for Myanmar
313	*Yakirra*	*Panicum*	
314	*Ypsilandra*	*Helonias*	
