# Supplementary material for: ﻿An updated checklist of vascular plants of Myanmar
Source: PhytoKeys. 2025 Aug 11;261:135–64. doi: 10.3897/phytokeys.261.154986 (PMC12361923; doi:10.3897/phytokeys.261.154986)
Supplement: Supplementary material 5 — List of references for Myanmar plant species data [file phytokeys-261-135_article-154986__-s005.pdf]

List of references for Myanmar plant species data

1. Aubriot X, Knapp S (2022) A revision of the "spiny solanums" of Tropical Asia (Solanum, the Leptostemonum Clade, Solanaceae). PhytoKeys 198: 1-270.
2. Aung MH, Jin X-H (2021) Phalaenopsis putaoensis (Orchidaceae: Epidendroideae, Vandeae), a new species from Kachin State, Myanmar. Phytotaxa 484: 244-246.
3. Aung MM, Tanaka N (2019) Seven Taxa of Zingiber (Zingiberaceae) Newly Recorded for the Flora of Myanmar. Bull Natl Mus Nat Sci, Ser B 45(1): 1–8.
4. Aung MM, Tanaka N, Miyake N (2015a) Larsenianthus arunachalensis (Zingiberaceae): A new distributional record for the flora of Myanmar. Rheedea 25(2): 94-96.
5. Aung MM, Tanaka N, Miyake N (2015b) Two Gingers, Zingiber orbiculatum and Z. flavomaculosum (Zingiberaceae), Newly Recorded from Myanmar. Bull Natl Mus Nat Sci, Ser B 41(3): 1–6.
6. Aung TS, Xu Z-P (2023) Update Checklist of Higher Plants in Myanmar (2023) Version 1.1. In: (CAS) CAoS (Ed). GBIF.
7. Aung YL, Jin X-H (2018) Gastrodia kachinensis (Orchidaceae), a new species from Myanmar. PhytoKeys 94: 23-29.
8. Aung YL, Jin X-H, Schuiteman A (2017) Coelogyne putaoensis (Orchidaceae), a new species from Myanmar. PhytoKeys 82: 27-34.
9. Aung YL, Mu AT, Aung MH, Jin X-H (2021) Field Guide to Orchids of Myanmar. Contributions from Southeast Asia Biodiversity Research Institute, CAS. Hubei Science and Technology Press, Wuhan, China, 248 pp.
10. Aung YL, Mu AT, Aung MH, Liu Q, Jin X-H (2020) An annotated checklist of Myanmar orchid flora. PhytoKeys 138: 49-112.
11. Aung YL, Mu AT, Jin X-H (2018) Odontochilus putaoensis (Cranichideae, Orchidaceae), a new species from Myanmar. PhytoKeys: 19-26.
12. de Kok R (2012) A revision of the genus Gmelina (Lamiaceae). Kew Bulletin 67: 293-329.
13. Dickason FG (1946) The Ferns of Burma. In.
14. Ding H-B, Bin Y, Zhou S-S, Li R, Maw MB, Kyaw WM, Tan Y-H (2018) Hedychium putaoense (Zingiberaceae), a new species from Putao, Kachin State, Northern Myanmar. PhytoKeys 94: 51-57.
15. Ding H-B, Yang B, Maw MB, Win PP, Tan Y-H (2020) Taxonomic studies on Amomum Roxburgh s.l. (Zingiberaceae) in Myanmar II: one new species and five new records for the flora of Myanmar. PhytoKeys 138: 139-153.
16. Ding H-B, Yang B, Zhou S-S, Maw MB, Maung KW, Tan Y-H (2019a) New contributions to the flora of Myanmar I. Plant Diversity 41: 135-152.
17. Ding H-B, Zhou S-S, Yang B, Li R, Maw MB, Maung KW, Tan Y-H (2019b) Taxonomic studies on Amomum Roxburgh s.l. (Zingiberaceae) in MyanmarI: Two new species and two new records for the flora of Myanmar. Phytotaxa 418: 158-170.
18. Do TV, Li J-W (2018) Aristolochia bhamoensis sp. nov. (Aristolochiaceae) and a diagnostic key to all known A. subgen. Siphisia species from Myanmar. Nordic Journal of Botany 36: e01909.
19. Dong S-Y (2020) Synopsis of Cyatheaceae from Myanmar. Phytotaxa 449: 207-216.
20. Floden A, Nwe TY, Armstrong KE (2023) A New Polygonatum, P. bifolium species (Asparagaceae: Nolinoideae: Polygonateae) from Myanmar and a Checklist of the Species Known to Occur There. Novon 31: 169-177.

21. Fritsch PW, Armstrong KE, Aung MM, Fujikawa K, Lu LU (2023) Gaultheria (Ericaceae) of Myanmar: an updated species list for the country, a new species, and a new species combination. Phytotaxa 595: 037-061.
22. Fujikawa K, Baba Y, Shin T, Moe AZ, Mizukami H (2021a) Taxonomic Enumeration of Natma Taung National Park Vol. 1. Kobun Printing Co., Ltd., Kochi, Japan.
23. Fujikawa K, Baba Y, Shin T, Moe AZ, Mizukami H (2021b) Taxonomic Enumeration of Natma Taung National Park Vol. 2. Kobun Printing Co., Ltd., Kochi, Japan.
24. Fujiwara T, Khine PK, Hori K, Shin T, Murakami N, Schneider H (2022) Lepisorus medioximus (Polypodiales, Polypodiaceae), a new species from Shan State of Myanmar. PhytoKeys 201: 23-34.
25. Gong Q-B, Landrein S, Xi H-C, Ma X-D, Yang Z-H, He K-W, Shen J-Y (2018) Aristolochia tongbiguanensis, a new species of Aristolochiaceae from Yunnan, China. Taiwania 63: 183-187.
26. Hein KZ, Naive MAK (2021) Taxonomic studies of Araceae in Myanmar III: Typhonium aungmyintwinii, a new species from Mogok Township, Mandalay Region. Taiwania 66: 455-458.
27. Hein KZ, Naive MAK (2024) Taxonomic studies of Araceae in Myanmar VII: Two new species and a new record for the genus Homalomena. Journal of Asia-Pacific Biodiversity 17: 343-350.
28. Hein KZ, Naive MAK, Chen J (2021) Artabotrys chitkokoi (Annonaceae), a new species discovered in Sagaing Region, Myanmar. Nordic Journal of Botany 39.
29. Hein KZ, Naive MAK, Serebryanyi MM (2022) Taxonomic studies of Araceae in Myanmar V: a new Alocasia species from Myanmar and Thailand. Nordic Journal of Botany 2022.
30. Hein KZ, Saensouk S, Naive MAK (2024) Taxonomic studies of Araceae in Myanmar VIII: Two new species and three new records for the genus Typhonium. Taiwania 69: 537-544.
31. Herrando-Moraira S, Group TCR, Calleja J-A, Chen Y-S, Fujikawa K, Galbany-Casals M, Garcia-Jacas N, Kim S-C, Liu J-Q, López-Alvarado J, López-Pujol J, Mandel JR, Mehregan I, Roquet C, Sennikov AN, Susanna A, Vilatersana R, Xu L-S (2020) Generic boundaries in subtribe Saussureinae (Compositae: Cardueae): Insights from Hyb-Seq data. TAXON 69: 694-714.
32. Hori K, Ebihara A, Moe AZ (2019) New Records of Pteridophytes from Myanmar (1). J Jpn Bot 94(1): 35–38.
33. Hori K, Khine PK, Fujiwara T, Shin T, Schneider H (2022a) Thylacopteris minuta (Polypodiaceae), a new fern species from Myanmar. PhytoKeys: 141-153.
34. Hori K, Moe AZ (2019) Hymenasplenium quangnamense (Aspleniaceae) New to Myanmar. J Jpn Bot 94(5): 321–324.
35. Hori K, Moe AZ (2020) Pterygiella nigrescens (Orobanchaceae), New to Myanmar. Acta Phytotaxonomica et Geobotanica 71: 249-254.
36. Hori K, Schneider H, Khine PK (2022b) Taxonomic Enumeration of ferns and lycophytes from Popa, Mandalay Region in Myanmar. Phytotaxa 558: 53-66.
37. Hughes M, Aung MM, Armstrong K (2019) An Updated Checklist and a New Species of Begonia (B. Rheophytica) from Myanmar. Edinburgh Journal of Botany 76: 285-295.

38. Hundley HG (1987) List of Trees, Shrubs, Herbs and Principal Climbers, etc. Recorded from Burma with Vernacular Names. 4th Revised Ed. Forest Department, Swe Daw Oo Press, Rangoon.
39. Hundley HG, Ko CK (1961) List of Trees, Shrubs, Herbs and Principal Climbers, etc. Recorded from Burma with Vernacular Names. 3rd Ed. Supdt., Govt. Printing and Staty., Rangoon.
40. Ito Y, Barfod A (2014) An updated checklist of aquatic plants of Myanmar and Thailand. Biodiversity Data Journal 2.
41. Ito Y, Ohi-Toma T, Tanaka N, Murata J (2009) New or Noteworthy Plant Collections from Myanmar (3) Caldesia parnassifolia, Nechamandra alternifolia, Potamogeton maackianus and P. octandrus. J Jpn Bot 84: 321–329.
42. Ito Y, Tanaka N, Pooma R, Tanaka N (2014) DNA barcoding reveals a new record of Potamogeton distinctus (Potamogetonaceae) and its natural hybrids, P. distinctus × P. nodosus and P. distinctus × P. wrightii (P. ×malainoides) from Myanmar. Biodiversity Data Journal 2.
43. Jin X-H, Kyaw M (2017) Gastrodia putaoensis sp. nov. (Orchidaceae, Epidendroideae) from North Myanmar. Nordic Journal of Botany 35: 730-732.
44. Jin X-H, Mint Z (2018) Petrosaviaceae, a new familial record of angiosperm from Myanmar. Redai Yaredai Zhiwu Xuebao 26(6): 677-678.
45. Jin X-H, Zhu X-Y, Mint Z (2018) Triuridaceae, a new familial record of Angiosperm from Myanmar. Redai Yaredai Zhiwu Xuebao 26(1): 79-81.
46. Jung E-K, Kang D-H, Ong HG, Kyaw NO, Kim Y-D (2024) New records of flowering plants for the flora of Myanmar collected from Southern Shan State II. Korean Journal of plant Taxonomy 54: 139-143.
47. Kang D-H, Kyaw NO, Jung E-K, Shin J-S, Kim Y-D, Ong HG (2018) New records of flowering plants for the flora of Myanmar collected from southern Shan State. Korean Journal of plant Taxonomy 48: 218-229.
48. Kang D-H, Ling SM, Kim Y-D, Ong HG (2017) New records of flowering plants of the flora of Myanmar collected from Natma Taung National Park (Chin State). Korean Journal of plant Taxonomy 47: 199-206.
49. Kang D-H, Ong HG, Lee J-H, Jung E-K, Kyaw N-O, Fan Q, Kim Y-D (2021) A new broad-leaved species of loquat from eastern Myanmar and its phylogenetic affinity in the genus Eriobotrya (Rosaceae). Phytotaxa 482: 279-290.
50. Kazumi F, Hiroshi I, Ling Shein M, Law S (2017) Taxonomic Studies in the Asteraceae of Myanmar III: A New Species, Ainsliaea hederifolia, from Myanmar. The Journal of Japanese Botany 92: 87-93.
51. Kazumi F, Hiroshige K (2008) Ligularia japonica Less. (Asteraceae), a New Record for Myanmar. The Journal of Japanese Botany 83: 356-358.
52. Khine PK, Fraser-Jenkins C, Lindsay S, Middleton D, Miehe G, Thomas P, Kluge J (2017) A Contribution Toward the Knowledge of Ferns and Lycophytes from Northern and Northwestern Myanmar. American Fern Journal 107: 219-256.
53. Khine PK, Lindsay S, Fraser-Jenkins C, Kluge J, Kyaw M, Hovenkamp P (2016) Selliguea kachinensis (Polypodiaceae), a new fern species of uncertain affinity from Northern Myanmar. PhytoKeys 62: 73-81.

54. Khine PK, Schneider H (2020) Data on pteridophyte species diversity and status of the International Union for Conservation of Nature in each political unit of Myanmar. Data in Brief 30: 105503.
55. Koopowitz H, Iamwiriyakul P, Laohapatcharin S (2017) Paphiopedilum myanmaricum, a new species of slipper orchid (Cypripedioideae, Orchidaceae). Phytotaxa 324.
56. Koyama T (2018) Notes on Cyperaceae from Myanmar. Part 2. Taiwania 63: 1-6.
57. Kress WJ, DeFilipps RA, Farr E, Kyi YY (2003) A checklist of the trees, shrubs, herbs, and climbers of Myanmar (revised from the original works by JH Lace, R. Rodger, HG Hundley and U Chit Ko Ko on the "List of trees, shrubs, herbs and principal climbers etc. recorded from Burma"). Contributions from the United States National Herbarium. 590 pp.
58. Kurzweil H (2013) Calanthe punctata (Orchidaceae), a new species from southern Myanmar. Gardens' Bulletin Singapore 65(2): 163–168.
59. Kurzweil H, Lwin S (2014) A guide to orchids of Myanmar. Natural History Publications (Borneo), Kota Kinabalu.
60. Kurzweil H, Ormerod P (2018) Precursory studies on the orchid flora of Myanmar with one new species and thirty eight new distribution records. Rheedea 28.
61. Kurzweil H, Ormerod P (2019) A review of the Calanthe group (Orchidaceae) in Myanmar. Thai Forest Bulletin (Botany) 47: 196-225.
62. Kurzweil H, Ormerod P, Kumar S (2023) The genus Habenaria (Orchidaceae) in Myanmar. Thai Forest Bulletin (Botany) 51: 98-156.
63. Kurzweil H, Ormerod P, Schuiteman A (2020) The long-lost Myanmar endemic Arundina subsessilis (Orchidaceae) found congeneric with the recently described Chinese Thuniopsis cleistogama. Gardens' Bulletin Singapore 72: 97-107.
64. Kurzweil H, Tanaka N, Aung MM, Ormerod P (2022) A new species of Luisia (Orchidaceae) from Shan State, Myanmar. Gardens' Bulletin Singapore.
65. Kurzweil H, Watthana S, Lwin S (2010) Phaius takeoi (Orchidaceae) Newly Recorded from Thailand and Myanmar. The Gardens' Bulletin, Singapore 62: 105-110.
66. Lace JH (1912) List of Trees, Shrubs, Herbs and Principal Climbers, etc. Recorded from Burma. Forest Department, CCF, Rangoon.
67. Latt MM, Naing MK, Choudhary RK, Lee J (2024) A new species of Globba under Sect. haplanthera (Zingiberaceae) from Myanmar. Bangladesh Journal of Plant Taxonomy 31: 197-203.
68. Latt MM, Park BB, Tanaka N (2020) Dendrobium calicopis (Orchidaceae), a New Record for Myanma. Bull Natl Mus Nat Sci, Ser B, 46(3): 107–110.
69. Latt MM, Park BB, Tanaka N (2021) Nervilia cumberlegei (Orchidaceae), a Newly Recorded Orchid from Myanmar. Bull Natl Mus Nat Sci, Ser B 47(1): 41–44.
70. Latt MM, Tanaka N, Park BB (2023) Two New species of Impatiens (Balsaminaceae) from Myanmar. Phytotaxa 583.
71. Leong-Škorničková J, Šída O, Záveská E, Marhold K (2015) History of infrageneric classification, typification of supraspecific names and outstanding transfers in Curcuma (Zingiberaceae). TAXON 64: 362-373.
72. Li H-M, Ren C (2018) Cissampelopsis quinquesquamata (Asteraceae–Senecioneae), a new species from western Myanmar and northeastern India. Nordic Journal of Botany 36: e01977.

73. Li J-W, Tan Y-H, Wang X-L, Wang C-W, Jin X-H (2018) Begonia medogensis, a new species of Begoniaceae from Western China and Northern Myanmar. PhytoKeys 103: 13-18.
74. Li L, Tan Y-H, Meng H-H, Ma H, Li J (2020) Two new species of Alseodaphnopsis (Lauraceae) from southwestern China and northern Myanmar: evidence from morphological and molecular analyses. PhytoKeys 138: 27-39.
75. Li L, Ye D-P, Niu M, Yan H-F, Wen T-L, Li S-J (2015) Thuniopsis: A New Orchid Genus and Phylogeny of the Tribe Arethuseae (Orchidaceae). Plos One 10: e0132777.
76. Liu Q, Zhou S-S, Jin X-H, Pan B, Maung KW, Zyaw M, Li R, Quan R-C, Tan Y-H (2018) Dendrobium naungmungense (Orchidaceae, Dendrobieae), a new species from Kachin State, Myanmar. PhytoKeys 94: 31-38.
77. Liu Q, Zhou S-S, Li R, Zhang M-X, Zyaw M, Lone S, Quan R-C (2017) Bulbophyllum putaoensis (Orchidaceae: Epidendroideae; Malaxideae), a new species from Kachin State, Myanmar. Phytotaxa 305: 57-60.
78. Maw MB, Ding H-B, Yang B, Win PP, Tan Y-H (2020) Taxonomic studies on Begonia (Begoniaceae) in Myanmar I: three new species and supplementary description of Begonia rheophytica from Northern Myanmar. PhytoKeys 138: 203-217.
79. Maw MB, Ding H-B, Yang B, Wino PP, Tan Y-H (2021) Taxonomic studies on Begonia (Begoniaceae) in Myanmar II: seven new species from Myanmar. Taiwania 66: 214-231.
80. Maw MB, Hein KZ, Naing MK, Yu W-B, Tan Y-H (2023) Taxonomic studies on Begonia (Begoniaceae) in Myanmar III: Begonia kayinensis (sect. Monophyllon), a remarkable new species from Kayin State, Southern Myanmar. Taiwania 68: 407-411.
81. Mood JD, Tanaka N, Aung MM, Murata J (2016) The genus Boesenbergia (Zingiberaceae) in Myanmar with two new records. Gardens' Bulletin Singapore 68.
82. Mu A-T, Aung M-H, Jin X-H (2020) Neottia nyinyikyawii (Orchidaceae: Epidendroideae), a new species from Chin State, Myanmar. Phytotaxa 446: 205-208.
83. Mu AT, Aung YL, Jin X (2019) Liparis popaensis (Orchidaceae), a new species from Myanmar. Phytotaxa 413: 67-70.
84. Murata J, Aung MM, Tanaka N (2020a) Contributions to the Flora of Myanmar VI: Arisaema kayahense (Araceae), a New Species from Kayah State. J Jpn Bot 95(2): 85–88.
85. Murata J, Ohi-Toma T, Aung MM, Tanaka N (2020b) Taxonomy of Hirsutiarum (Araceae), a New Genus Record for the Flora of Myanmar. J Jpn Bot 95(5): 285–290.
86. Murata J, Ohi-Toma T, Tanaka N (2010) New or Noteworthy Plant Collections from Myanmar (4): Typhonium cordifolium and two new species, T. neogracile and T. praecox (Araceae). J Jpn Bot 85: 1–7.
87. Nagahama A, Sugawara T, Aung MM, Poulsen AD, Armstrong KE, Tagane S, Tanaka N (2023) Contributions to the Flora of Myanmar IX: Five New Distributional Records of Flowering Plants from Chin State, Kachin State and Tanintharyi Region. Bulletin of the National Museum of Nature and Science Series B, Botany 49: 49-55.
88. Naive MAK, Hein KZ (2020) Lost and found: Rediscovery of Ruellia bella Craib (Acanthaceae) after over a century, and first record of this species in Myanmar. Feddes Repertorium 131: 278-282.
89. Naive MAK, Hein KZ (2021) Taxonomic studies of Araceae in Myanmar II: Typhonium edule, a remarkable new aroid species from Monywa District, Sagaing Region. Phytotaxa 513: 159-165.

90. Naive MAK, Hein KZ, Hetterscheid W (2022a) Taxonomic studies of Araceae in Myanmar IV: A new species, a new record and a new synonym for the genus Amorphophallus. Blumea 67: 123-128.
91. Naive MAK, Hein KZ, Kumar P, Ormerod P (2022b) Eulophia myanmarica (Orchidaceae), a new species from Myanmar and notes on Geodorum densiflorum. Phytotaxa 548: 288-294.
92. Naive MAK, Hein KZ, Serebryanyi M, Hetterscheid W (2024) Taxonomic studies of Araceae in Myanmar VI: Amorphophallus mirabilis - a new species and a new record for the genus Amorphophallus. Nordic Journal of Botany 2024.
93. Nwe TY, Moon M-O, Lee S-H, Sun B-Y (2019) Newly recorded ferns from the flora of Myanmar in Natma Taung National Park. Korean Journal of plant Taxonomy 49: 8-12.
94. Ohi-Toma T, Watanabe-Toma K, Aung MM, Tanaka N, Murata J (2021) Contributions to the Flora of Myanmar VIII: Aristolochia kachinensis, a New Species of Subgenus Siphisia (Aristolochiaceae) from Kachin State. J Jpn Bot 96(6): 315–320.
95. Ormerod P, Kurzweil H (2020) Pinalia taunggyiensis (Orchidaceae), a New Species from Myanmar. Harvard Papers in Botany 25.
96. Ormerod P, Kurzweil H, Truong BV (2022) Additional Notes on the Orchid Flora of Myanmar and Some Other Ancillary Studies. Harvard Papers in Botany 27: 61-74, 14.
97. Ormerod P, Kurzweil H, Watthana S (2021) Annotated List of Orchidaceae for Myanmar. Phytotaxa 481: 1-262.
98. Ormerod P, Wood EW (2010) A New Species of Pinalia (Orchidaceae: Eriinae) from Myanmar. Harvard Papers in Botany 15: 349-351.
99. Paing CS, Suksathan P, Ruchisansakun S (2024) Impatiens karenensis (Balsaminaceae), a new tiny flowered species from Myanmar. PhytoKeys: 113-119.
100. Paszko B (2014) Deyeuxia himalaica (Poaceae, Agrostidinae): taxonomy and its first record from Myanmar. Phytotaxa 156: 285-290.
101. Pedersen HÆ, Suksathan P, Indhamusika S (2002) Sirindhornia, a new orchid genus from Southeast Asia. Nordic Journal of Botany 22: 391-404.
102. Peng C-I, Wang H, Kono Y, Yang H-A (2014) Begonia wui-senioris (sect. Platycentrum, Begoniaceae), a new species from Myanmar. Botanical Studies 55: 13.
103. Peng Y, Yang C, Luo Y (2020) Blumea htamanthii (Asteraceae), a new species from Myanmar. PhytoKeys 138: 225-232.
104. Rodda M, Armstrong K, Klackenberg J (2024) Apocynaceae of continental South-East Asia: new species, new records and new combinations. Thai Forest Bulletin (Botany): 5-20.
105. Rodda M, Aung MM, Armstrong K (2019) A new species, a new subspecies, and new records of Hoya (Apocynaceae, Asclepiadoideae) from Myanmar and China. Brittonia 71: 424-434.
106. Rodger A (1922) List of Trees, Shrubs, Herbs and Principal Climbers, etc. Recorded from Burma with Vernacular Names. (ed. 2). Forest Department, Rangoon.
107. Ruchisansakun S, Suksathan P, Van der Niet T, Saw L, Janssens SB (2017) Impatiens tanintharyiensis (Balsaminaceae), a new species from Southern Myanmar. Phytotaxa 296: 171-179.
108. Ruchisansakun S, Suksathan P, Van Der Niet T, Smets EF, Lwin SAW, Janssens SB (2018a) Three new species of Impatiens (Balsaminaceae) from Myanmar. Phytotaxa 338.

109. Ruchisansakun S, Suksathan P, van der Niet T, Smets EF, Saw L, Janssens SB (2018b) Balsaminaceae of Myanmar. Blumea - Biodiversity, Evolution and Biogeography of Plants.
110. Saensouk P, Saensouk S, Boonma T, Oo WP, Htet NM, Maknoi C, Bongcheewin B, Htway NN, Minn HM (2025) Two new records of Boesenbergia Kuntze (Zingiberaceae: Zingibereae) for the Flora of Myanmar. Biodiversitas Journal of Biological Diversity 26.
111. Shen SK, Ding HB, Tan YH (2024) Nothapodytes burmanica (Icacinaceae), a new species from Kachin State, Myanmar. Nordic Journal of Botany 2024.
112. Tagane S, Tanaka N, Aung MM, Naiki A, Yahara T (2018) Contributions to the Flora of Myanmar II: New records of eight woody species from Tanintharyi Region, Southern Myanmar. Nat Hist Bull Siam Soc 63(1): 47–56.
113. Tagane S, Toyama H, Tanaka N, Aung MM, Nagahama A, Win AK, Win SS, Yahara T (2019) Contributions to the flora of Myanmar III: new records of 10 woody species from the Mergui Archipelago of Southern Myanmar. NAT HIST BULL SIAM SOC 63(2): 141–151.
114. Tan Y-H, Li D-R, Zhou S-S, Chen Y-J, Bramley GLC, Li B (2018) Premna grandipaniculata (Lamiaceae, Premnoideae), a remarkable new species from north Myanmar. PhytoKeys 94: 117-123.
115. Tan Y-H, Min D-Z, Ding H-B, Yang B, Maw MB, Li B (2021) Premna caridantha (Lamiaceae: Permnoideae), a distinct new species from Kachin State, northern Myanmar. Phytotaxa 490: 107-113.
116. Tan Y-H, Yang B, Li J-W, Zhou S-S, Shwelone S, Khaing K, Li R, Huang J-P, Sun H (2015) Acranthera burmanica, a new species of Rubiaceae from Kachin State, Myanmar. Phytotaxa 238.
117. Tan Y, Li D, Chen Y, Li B (2017) Premna bhamoensis (Lamiaceae, Premnoideae), a new species from Kachin State, northeastern Myanmar. PhytoKeys 83: 93-101.
118. Tanaka N, Armstrong K, Aung MM, Naiki A (2020) Taxonomic studies on Zingiberaceae of Myanmar II: Curcuma stolonifera (Subgenus Ecomatae), a new species from the northwestern region. Brittonia 72: 268-272.
119. Tanaka N, Aung MM (2017) A new species of Zingiber (Sect. Dymczewiczia: Zingiberaceae) from northwestern Myanmar. Phytotaxa 316.
120. Tanaka N, Aung MM (2019) Taxonomic Studies on Zingiberaceae of Myanmar I: A new species of Curcuma (Subgenus Ecomatae) from Myanmar. Phytotaxa 387.
121. Tanaka N, Aung MM, Latt MM (2018a) Thismia breviappendiculata (Thismiaceae), a New Mycoheterotrophic Plant from Southern Myanmar. Bull Natl Mus Nat Sci, Ser B 44(2): 67–72.
122. Tanaka N, Aung MM, Vermeulen JJ (2022a) Impatiens katjae, a New Species of Impatiens (Balsaminaceae) from Central Myanmar. Novon 30: 56-60.
123. Tanaka N, Hayami Y (2011) Begonia kachinensis (Begoniaceae, sect.Sphenanthera), a New Species from Myanmar. Acta Phytotax Geobot 61 (3): 151–154.
124. Tanaka N, Hughes M (2007) Begoina (sect. Sphenanthera) hayamiana (Begoniaceae), a New Species from Northern Myanmar. Acta Phytotax Geobot 58(2/3): 83-86.
125. Tanaka N, Kadota Y, Murata J (2010a) New or Noteworthy Plant Collection from Myanmar (6): Ranunculaceae of Mt. Victoria, Chin State, Myanmar. J Jpn Bot 85: 199–212.

126. Tanaka N, Kobayashi S, Ohi-Toma T, Murata J (2006) New or Noteworthy Plant Collections from Myanmar (1): Hydrobryum japonicum, Balanophora subcupularis, Rhopalocnemis phalloides and Sonerila laeta. J Jpn Bot 81: 324-331.
127. Tanaka N, Miyake K, Aung MM (2022b) Taxonomic Studies on Zingiberaceae of Myanmar IV: A New Species of Zingiber (Sect. Cryptanthium) from Kayah State. Bulletin of the National Museum of Nature and Science Series B, Botany 48: 17-21.
128. Tanaka N, Nagamasu H (2006) A New Record of Gmelina tomentosa Fletcher (Verbenaceae)from Myanmar. Acta Phytotax Geobot 57 (3): 233-236.
129. Tanaka N, Nagamasu H, Tagane S, Aung MM, Win AK, Hnin PP (2019) Contributions to the Flora of Myanmar IV: A new species and a newly recorded taxon of the genus Sapria (Rafflesiaceae). Taiwania 64: 357-362.
130. Tanaka N, Ohi-Toma T, Murata H, Aung MM, Murata J (2016) New or Noteworthy Plant Collections from Myanmar (9) Agapetes (Ericaceae) from Northwestern Myanmar. J Jpn Bot 91 Suppl.: 99–111.
131. Tanaka N, Ohi-Toma T, Murata J (2009) A Taxonomic Study of Rosa clinophylla var. glabra (Rosaceae), Newly Recorded from Myanmar, Based on Morphological and Molecular Data. J Jpn Bot 84: 27–32.
132. Tanaka N, Ohi-Toma T, Murata J (2010b) A new species of Argostemma (Rubiaceae) from Mount Victoria, Myanmar. Blumea - Biodiversity, Evolution and Biogeography of Plants 55: 65-67.
133. Tanaka N, Ohi-Toma T, Suksathan P, Aung MM, Poulsen AD, Mohamad S, Armstrong EK (2022c) Myanmaranthus roseiflorus, a New Genus and Species of Marantaceae from Myanmar. J Jpn Bot 97(4): 187–196.
134. Tanaka N, Paing CS, Aung MM (2024) Taxonomic Studies of Zingiberaceae in Myanmar V: A New Species of Boesenbergia from Rakhine State. Bulletin of the National Museum of Nature and Science Series B, Botany 50: 149-152.
135. Tanaka N, Peng C-I (2016) Begonia togashii (Begoniaceae: Sect. Platycentrum), a New Species from Central Myanmar. Acta Phytotax Geobot 67(3): 191–197.
136. Tanaka N, Sugawara T, Aung MM, Murata JIN (2015a) Impatiens kingdon-wardii (Balsaminaceae), a new species from Mt. Victoria (Natma Taung), Myanmar. Phytotaxa 234.
137. Tanaka N, Tagane S, Naiki A, Aung MM, Tanaka N, Dey S, Mood J, Murata J (2018b) Contributions to the Flora of Myanmar I: Nine taxa of monocots newly recorded from Myanmar. Bull Natl Mus Nat Sci, Ser B 44(1): 31–39.
138. Tanaka N, Tanaka N, Ohi-Toma T, Murata J (2007) New or Noteworthy Plant Collections from Myanmar (2)Aponogeton lakhonensis, Cryptocoryne cruddasiana, C. crispatula var. balansae and Stichoneuron membranaceum. J Jpn Bot 82: 266–273.
139. Tanaka N, Tetsuo O-T, Ito Y, Aung MM, Jin M (2018c) New or Noteworthy Plant Collections from Myanmar (10): Impatiens hukaungensis (Balsaminaceae), a New Rheophyte from the Northern Region. J Jpn Bot 93(1): 23–30
140. Tanaka N, Tetsuo O-T, Tagane S, Kana W-T, Sugawara T, Aung MM, Jin M (2021) Contributions to the Flora of Myanmar VII: Nine new distributional records of flowering plants from Myanmar. THAI JOURNAL OF BOTANY 13 (1): 47–57.
141. Tanaka N, Yukawa T, Htwe KM, Murata J (2015b) An Orchid Checklist of Mt. Popa, Central Myanmar. Bull Natl Mus Nat Sci, Ser B 41(2): 69–89.

142. Tanaka N, Yukawa T, Murata J (2010c) New or Noteworthy Plant Collections from Myanmar (5) : Dendrobium koyamae, a New Species in sect. Formosa (Orchidaceae). Acta Phytotax Geobot 60 (3): 171-174
143. Tong Y-H, Fritsch PW, Tan Y-H, Aung MM, Yang B, Armstrong KE (2022) Novelties in Myanmar Agapetes (Ericaceae) with an updated checklist of species from the country. Nordic Journal of Botany 2022: e03496.
144. Toyama H, Aung MM, Tagane S, Naiki A, Suddee S, Nagamasu H, Nagahama A, Win SS, Tanaka N, Yahara T (2020) Contributions to the Flora of Myanmar V: a new record of Mallotus tokiae (Euphorbiaceae) with the description of flower morphology from Lampi Island. Thai Forest Bulletin (Botany) 48: 1-6.
145. Traiperm P, Fujikawa K, Chitchak N, Srisanga P, Maknoi C, Staples G (2019) A new species of Argyreia (Convolvulaceae) from Myanmar. Willdenowia 49: 65-70, 66.
146. Tseng Y-H, Kim Y-D, Peng C-I, Htwe KM, Cho S-H, Kono Y, Chung K-F (2017) Begonia myanmarica (Begoniaceae), a new species from Myanmar, and molecular phylogenetics of Begonia sect. Monopteron. Botanical Studies 58: 21.
147. Wahlsteen E (2019) A new species of Begonia L. (Begoniaceae) and some notes on Begonia difformis from Kachin, northern Myanmar. Phytotaxa 420: 241-248.
148. Watthana S, Fujikawa K, Kertsawang K (2015) First Record of Cremastra Lindl. (Orchidaceae) in Myanmar. In.
149. Wojtas Kp, Bandara C, Kumar P (2023) A new species of Chiloschista (Orchidaceae, Aeridinae) from Myanmar. Phytotaxa 612: 57-66.
150. Wojtas KP, Bandara C, Kumar P (2024) A new species of Holcoglossum (Orchidaceae, Aeridinae) from Southern Shan State, Myanmar with taxonomic notes on Holcoglossum himalaicum. Phytotaxa 638: 257-267.
151. Wood JR, Aung MM, Wells T, Armstrong KE (2022) Strobilanthes Blume (Acanthaceae) in Myanmar, a new species and an updated checklist. Kew Bulletin 77: 521-539.
152. Xu W-B, Liu Y-L, Yan J-J, Shen J-Y (2022) Marsdenia burmanica (Apocynaceae, Asclepiadoideae), a new species from Kachin, Myanmar. Taiwania 67: 450-454.
153. Ya J-D, Jin X-H, Liu C (2019) Two new species of Cylindrolobus (Orchidaceae) from the eastern Himalayas. PhytoKeys 130: 107-113.
154. Yang B, Deng M, Zhang M-X, Moe AZ, Ding H-B, Maw MB, Win PP, Corlett RT, Tan Y-H (2020) Contributions to the flora of Myanmar from 2000 to 2019. Plant Diversity 42: 292-301.
155. Yang B, Ding H-B, Zhou S-S, Maw MB, Maung KW, Tan Y-H (2019a) Taxonomic studies on Agapetes in Myanmar I: Agapetes reflexiloba, a new species from Kachin State, and notes on three rediscovered species including two new records for Myanmar. Phytotaxa 393.
156. Yang B, Ding HB, Zhou SS, Zhu X, Li R, Maw MB, Tan YH (2018a) Aristolochia sinoburmanica (Aristolochiaceae), a new species from north Myanmar. PhytoKeys: 13-22.
157. Yang B, Zhou S-S, Liu Q, Win Maung K, Tan Y (2017a) Coelogyne magnifica (Orchidaceae), a new species from northern Myanmar. PhytoKeys 88: 109-117.
158. Yang B, Zhou S-S, Maung KW, Tan Y-H (2017b) Reinwardtia glandulifera (Linaceae), a new species from Kachin State, northern Myanmar. Phytotaxa 316.
159. Yang B, Zhou S-S, Maung KW, Tan Y-H (2017c) Two new species of Impatiens (Balsaminaceae) from Putao, Kachin State, northern Myanmar. Phytotaxa 321: 103-113.

160. Yang B, Zhou S-S, Tan Y-H (2019b) Canthium longipetalum (Rubiaceae), a New Species from Kachin State, Northern Myanmar. Annales Botanici Fennici 56.
161. Yang B, Zhou S, Ding H, Li R, Maung KW, Tan Y-H (2018b) Two new species of Trivalvaria (Annonaceae) from northern Myanmar. PhytoKeys 94: 1-10.
162. Yasushi I, Monthon N, Kazumi F (2017) Arthraxon microphyllus (Poaceae), a New Record for the Flora of Myanmar. The Journal of Japanese Botany 92: 62-66.
163. Yukawa T, Takamiya T, Aung MM, Htwe KM, Tanaka N (2022) Dendrobium popaense (Orchidaceae)—A New Species from Myanmar. Bulletin of the National Museum of Nature and Science Series B, Botany 48: 71-77.
164. Yukawa T, Tanaka N, Murata J (2010) Doritis natmataungensis (Orchidaceae), a New Species from Myanmar. Acta Phytotaxonomica et Geobotanica 60: 167-170.
165. Zhou S-S, Tan Y-H, Li REN, Quan R-C, Maung KW, Liu Q, Sima Y-K (2018a) Magnolia kachinensis (Magnoliaceae), a new species from northern Myanmar. Phytotaxa 375.
166. Zhou S-s, Tan Y, Jin X-h, Kyaw WM, Zyaw M, Li R, Quan R-C, Liu Q (2018b) Coelogyne victoria-reginae (Orchidaceae, Epidendroideae, Arethuseae), a new species from Chin State, Myanmar. PhytoKeys 98: 125-133.
167. Zhou S-S, Yang BIN, Tong Y-H, Ding H-B, Li REN, Kyaw WM, Tan Y-H (2017) Agapetes brevipedicellata (Ericaceae), a new species from Putao, Kachin State, Northern Myanmar. Phytotaxa 331.
168. Zhu X-X, Liao S, Tan Y-H, Shen J-Y, Ma J-S (2019) Aristolochia bhamoensis is a taxonomic synonym of A. tongbiguanensis, and now the correct name is Isotrema tongbiguanense. Phytotaxa 404.
